# Defects in placental syncytiotrophoblast cells are a common cause of developmental heart disease

**DOI:** 10.1038/s41467-023-36740-5

**Published:** 2023-03-01

**Authors:** Bethany N. Radford, Xiang Zhao, Tali Glazer, Malcolm Eaton, Danielle Blackwell, Shuhiba Mohammad, Lucas Daniel Lo Vercio, Jay Devine, Tali Shalom-Barak, Benedikt Hallgrimsson, James C. Cross, Henry M. Sucov, Yaacov Barak, Wendy Dean, Myriam Hemberger

**Affiliations:** 1 Dept. of Biochemistry and Molecular Biology, Cumming School of Medicine, Alberta Children’s Hospital Research Institute, University of Calgary, 3330 Hospital Drive NW, Calgary, AB T2N 4N1 Canada; 2 Dept. of Cell Biology and Anatomy, Cumming School of Medicine, Alberta Children’s Hospital Research Institute, University of Calgary, 3330 Hospital Drive NW, Calgary, AB T2N 4N1 Canada; 3 Magee-Women’s Research Institute, Dept. of Obstetrics/Gynecology and Reproductive Sciences, University of Pittsburgh, 204 Craft Ave., Pittsburgh, PA 15213 USA; 4 Dept. of Comparative Biology and Experimental Medicine, Faculty of Veterinary Medicine, Alberta Children’s Hospital Research Institute, University of Calgary, 3330 Hospital Drive NW, Calgary, AB T2N 4N1 Canada; 5 Dept. of Regenerative Medicine and Cell Biology, Division of Cardiology, Dept. of Medicine, Medical University of South Carolina, 173 Ashley Ave., Charleston, SC 29403 USA

**Keywords:** Embryogenesis, Development, Congenital heart defects

## Abstract

Placental abnormalities have been sporadically implicated as a source of developmental heart defects. Yet it remains unknown how often the placenta is at the root of congenital heart defects (CHDs), and what the cellular mechanisms are that underpin this connection. Here, we selected three mouse mutant lines, *Atp11a*, *Smg9* and *Ssr2*, that presented with placental and heart defects in a recent phenotyping screen, resulting in embryonic lethality. To dissect phenotype causality, we generated embryo- and trophoblast-specific conditional knockouts for each of these lines. This was facilitated by the establishment of a new transgenic mouse, *Sox2*-Flp, that enables the efficient generation of trophoblast-specific conditional knockouts. We demonstrate a strictly trophoblast-driven cause of the CHD and embryonic lethality in one of the three lines (*Atp11a*) and a significant contribution of the placenta to the embryonic phenotypes in another line (*Smg9*). Importantly, our data reveal defects in the maternal blood-facing syncytiotrophoblast layer as a shared pathology in placentally induced CHD models. This study highlights the placenta as a significant source of developmental heart disorders, insights that will transform our understanding of the vast number of unexplained congenital heart defects.

## Abstract (web summary)

Placental dysfunction can affect heart development, but the prevalence of this causality has not been well established. Here, the authors use mouse genetic tools to show that the placenta may constitute a significant source of congenital heart defects.

## Introduction

Congenital heart defects (CHDs) are by far the most common type of birth defect in humans, affecting ~1% of live births and accounting for over 10% of spontaneous miscarriages and still births^1,2^. Despite this prevalence, most CHDs remain of unknown etiology^3–6^. The search for genetic causes of disease usually focusses on the affected tissue itself, in this case the heart. While such studies have identified a number of key players directly involved in cardiac development^7^, a genetic explanation remains elusive for over 50% of CHD cases^3–6^.

Heart defects have been found to often co-occur with abnormalities in the placenta^8,9^. Commonly held views explaining this frequent co-occurrence include shared gene(s)/gene networks required for tissue development, as well as shared vascular abnormalities that may affect the heart and placenta alike^10^. However, in the mouse, it has been demonstrated that placental defects can also be the primary cause of some CHDs, often leading to a thinning of the myocardial wall and ventricular septal defects (VSDs)^11–19^.

These insights usually derive from experimental approaches whereby the mutant mouse embryo is provided with a functional placenta. In this scenario, the rationale is that if the heart defect is ameliorated, it must have originated from a dysfunctional placenta. Pinpointing proof of causality to the placental trophoblast lineage by ablating gene function only in trophoblast cells and observing a CHD as a consequence is pursued extremely rarely^17^. Moreover, these genetic rescue experiments have remained limited to few individual studies targeted towards specific genes of interest; a more systematic approach aimed at determining the frequency of placental defect-induced cardiac pathologies is missing, even in large-scale mouse mutant phenotyping efforts^20–24^.

By systematically including the placenta in phenotype assessments, it was recently determined that the vast majority of embryonic lethal mouse mutants exhibit placental dysmorphologies^8^. This work also revealed a close co-association of placental and heart phenotypes, but the cause-effect relationship remained unknown.

In the current study, we build on these data with the aim of gaining an unbiased view of how often heart defects are caused by placental dysfunction. To this end, we selected three mouse mutant lines on the basis of their fully penetrant placenta and heart phenotypes at embryonic day (E) 14.5, which is a developmental time point relevant to CHDs in human pregnancy. By using an epiblast-specific Cre driver^25^ and by establishing a novel genetic tool, a *Sox2*-Flp transgenic mouse that can be applied to the common knockout (KO)-first KOMP/EUCOMM targeted alleles^26^ to restore gene function in the entire epiblast, we generated embryo-specific and trophoblast-specific conditional KOs (cKOs), respectively, for each of the three lines. Our data demonstrate that the dysfunctional placenta is fully or partly responsible for the heart phenotype in two of these three lines. Importantly, we identify defects in the syncytiotrophoblast-I (SynT-I) layer as the primary placental abnormality that is associated with developmental heart pathologies, including in the *Pparg* mutation in which the placenta-heart axis paradigm was first established^11^. Moreover, gene expression signatures of SynT-I cells are indicative of heart abnormalities. Our study reveals the placenta as a significant source of developmental heart defects and highlights the critical importance of considering this organ in the etiology of CHDs.

## Results

### Mouse line selection

**Fig. 1:**
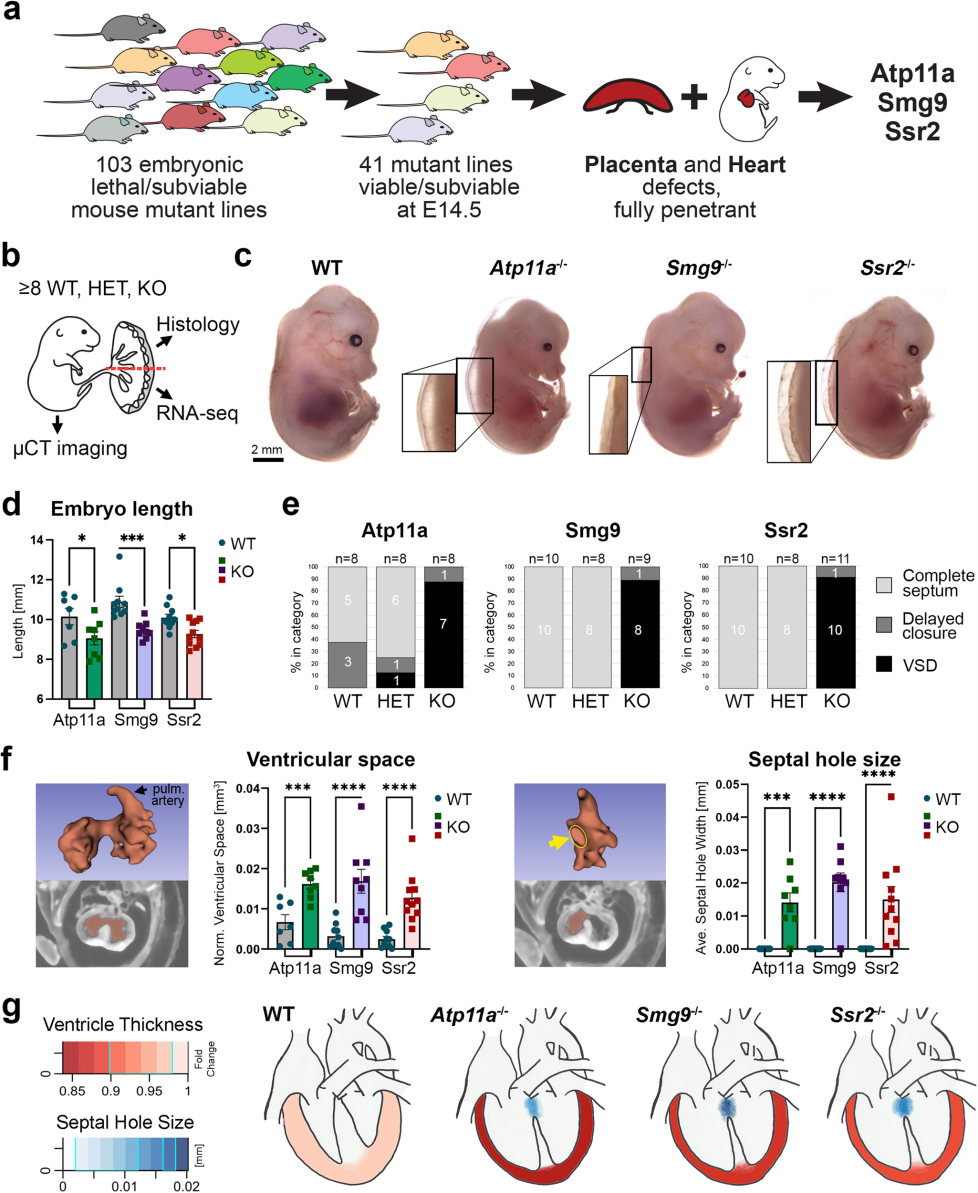
Mouse models of heart and placental failure. **a** Diagram depicting the rationale for the selection of mouse models from the DMDD cohort^8,27^ that suffer from cardiac and placental abnormalities with complete penetrance, and minimal defects in other organs. **b** Strategy for the comprehensive analysis of embryos and their associated placentas derived from tm1a/+ knockout-first allele intercrosses. The rendering of the embryo-placenta schematic is from Perez-Garcia, et al. Placentation defects are highly prevalent in embryonic lethal mouse mutants. Nature 555, 463-468 (2018). **c** E14.5 wild-type (WT) embryos and those mutant for *Atp11a*, *Smg9,* and *Ssr2*. Edema is frequently evident in mutant embryos of all three lines, highlighted in the magnified areas. Examples are representative of *n* = 8 embryos each. **d** Crown-rump length determined from µCT 3-dimensional (3D)-rendered scans. Knockout (KO) embryos of all three lines are significantly smaller. Data are displayed as mean ± SEM. Statistics: One-way ANOVA with Holm-Šídák’s multiple comparisons test. *p < 0.05, ***p < 0.001. **e** Assessment of overall heart morphology shows that all KO embryos suffer from ventricular septal defects (VSDs). Few *Atp11a* HET and WT embryos developed in heterozygous dams also exhibit mild abnormalities. Delayed septal closure was assigned when there was no overt connection between the ventricles but 1-2 image frames showed a very small hole in the high-resolution scans. **f** Segmentation analysis of 3D µCT imaging data for heart ventricular space volume and ventricular septal hole size (arrow). Ventricular space volume provides a measure inversely correlated to myocardial thinning. Severe VSDs are present in the majority of embryos analyzed. Data are displayed as mean ± SEM. One-way ANOVA, Holm-Šídák’s multiple comparisons test. ***p < 0.001, ****p < 0.0001. **g** Diagram depicting the VSDs and myocardial thinning consistently observed in all three mutant lines, color-coded for phenotype severity. Data in **d**–**g** is from *Atp11a*: WT *n* = 7, KO *n* = 8, *Smg9*: WT *n* = 10, KO *n* = 9; *Ssr2*: WT = 10, KO *n* = 11 samples. Source data are provided as a Source Data file. All exact *p* values are provided in Supplementary Data 1.

The ‘Deciphering the Mechanisms of Developmental Disorders’ (DMDD) project^27^ focused on the analysis of over 100 mouse KO lines that had an embryonic or perinatal lethal phenotype, in an effort aimed at unraveling the molecular framework that underpins embryo development. A particular novelty of the DMDD project was the systematic inclusion of the placenta in the phenotypic assessment, which revealed that placentation defects are highly prevalent in embryonic lethal mouse mutants^8^. By correlating embryonic and placental phenotypes it became evident that mouse mutant lines suffering from placental defects are disproportionately enriched for abnormal heart morphology^8^. Of the 41 mouse mutant lines assessed at E14.5 as part of DMDD, 18 (44%) displayed both, placental and heart defects. Here we selected three of these lines—*Atp11a*, *Smg9,* and *Ssr2*—based on the following criteria: (i) late-gestation or perinatal lethality to capture CHDs compatible with embryonic viability into the second half of gestation which is of relevance to human pregnancy and neonatal care needs, (ii) full penetrance of heart and placental defects, and (iii) limited defects in other embryonic organ systems, so as to minimize analytical complexity (Fig. 1a). All three genes are expressed in both, the placenta and the heart (Supplementary Fig. 1a–c), raising the possibility of either cardiac-specific effects or placental contributions to the heart phenotype. The three genes encode proteins with unrelated functions: ATP11A is a phospholipid flippase^28^, SMG9 is a component of the nonsense-mediated mRNA decay machinery^29^, and SSR2 is part of the translocon-associated protein (TRAP) complex responsible for transport of nascent proteins into the endoplasmic reticulum^30,31^. A linkage analysis found mutations in *SMG9* to be associated with preeclampsia as well as CHD in humans^32^. ATP11a and SSR2 have not been associated with human pregnancy complications or heart defects to date. Of note, none of these factors shares obvious functional pathway relationships with genes previously implicated in the placenta-heart axis.

### Evaluation of heart defects

The DMDD analysis comprised 3-6 KO embryos per line that often displayed substantial variability in phenotype severity and penetrance^8,33^. To gain more detailed insights into the mutant phenotypes of the three selected mouse lines, we initiated an in-depth analysis at E14.5 of at least 8 wildtype (WT), heterozygous (HET), and KO conceptuses per line, obtained by heterozygous intercrosses of the KO-first tm1a alleles (Supplementary Fig. 1d). The embryos were subjected to micro-computed tomography (µCT) imaging whereas the corresponding placentas were bisected for histological and transcriptomic analysis (Fig. 1b). This procedure enabled us to trace inter-individual variation on a phenotypic, morphological and molecular level.

KO embryos of all three genes were consistently smaller in size and exhibited varying degrees of edema, a common indicator of heart defects, in most but not all mutant conceptuses (Fig. 1c, d and Supplementary Table 1). We also noted that KO embryos had consistently smaller livers (Supplementary Fig. 1e), a phenotype that has been observed in other mutants that suffer from heart and/or placental defects^34,35^. All KO embryos exhibited a spectrum of cardiac defects (Supplementary Table 2); most notably, all homozygous KOs suffered from a failure in the closure of the ventricular septum (Fig. 1e, f), which is normally fully formed by E14.5^36^. In all cases, this resulted in a perimembranous VSD; in one *Ssr2* mutant, a muscular VSD was also observed (Supplementary Movies 1–3). This coincided with the presence of an overriding aorta (OA) or a double outflow right ventricle (DORV) phenotype in the vast majority of mutants (Supplementary Table 2). Moreover, all KOs displayed a pronounced thinning of the myocardial walls, including of the compact layer, as evidenced by the inversely correlated ventricular space volume µCT measurements (Fig. 1f and Supplementary Fig. 1f). This myocardial wall thinning was particularly obvious in the right ventricle, and was further confirmed on H&E-stained histological sections of mutant hearts (Supplementary Fig. 1g).

In the *Smg9* and *Ssr2* strains, all WT and HET embryos were overtly normal, including their hearts. For *Atp11a*, we observed a few HET and even WT embryos with a mild heart phenotype (Fig. 1e). This may point to a gene dosage-dependent phenotype of variable penetrance, possibly due to a maternal effect from the heterozygous dam. This notwithstanding, overall this analysis established that all three mouse mutant lines shared in common pronounced VSD and myocardial wall thinning phenotypes (Fig. 1g).

### Placental dysmorphologies

**Fig. 2:**
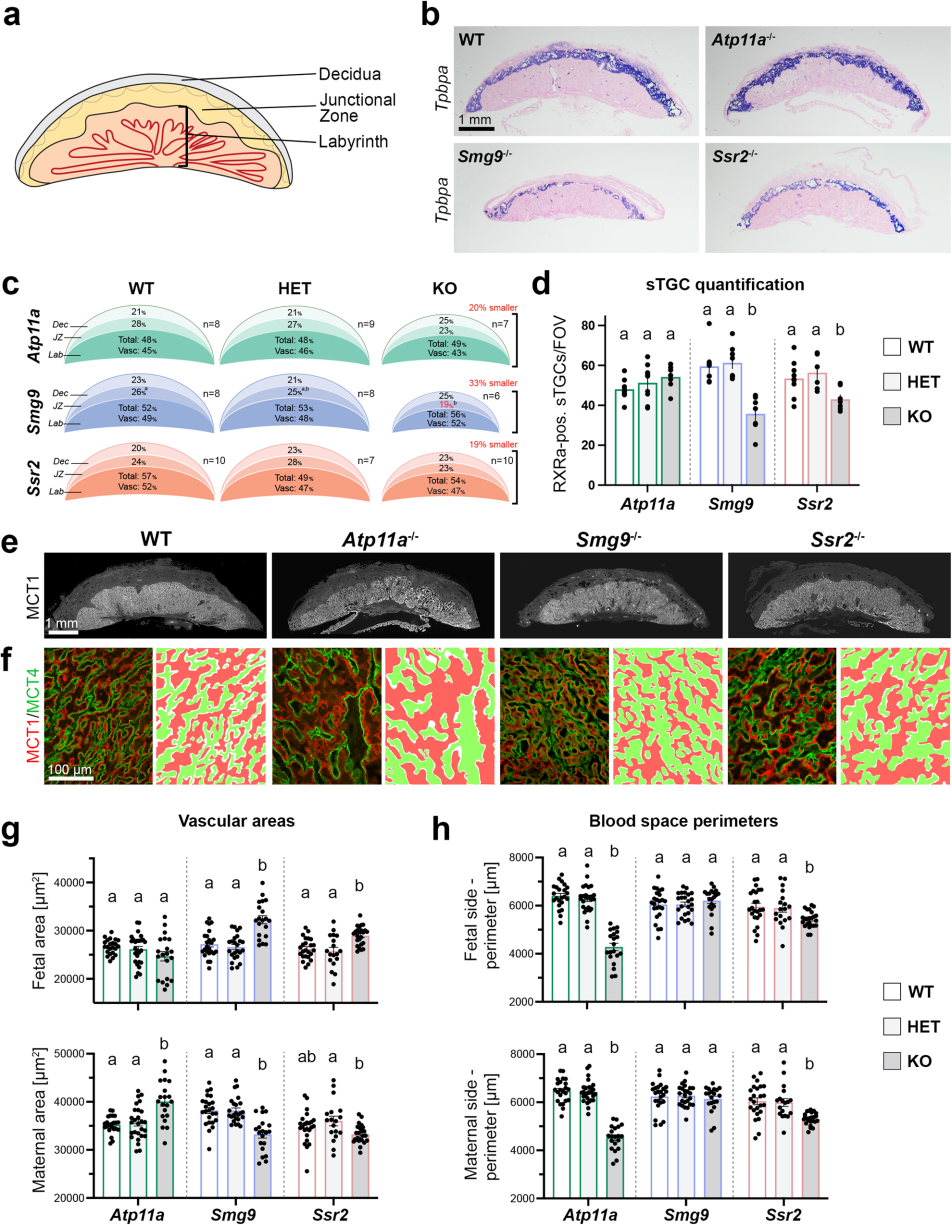
Analysis of placental defects in *Atp11a*, *Smg9* and *Ssr2* mutants. **a** Overview of the main structural areas of the mouse placenta. **b** *Tpbpa* in situ hybridization on wild-type (WT) and *Atp11a*^−/−^, *Smg9*^−/−^, and *Ssr2*^−/−^ placentas that were used to quantitate placental areas. Images are representative of *n* ≥ 6 samples per genotype. **c** Summary of the proportional area sizes in wild-type (WT), heterozygous (HET), and knockout (KO) placentas for each line. Placentas of all mutants were overall size-reduced. Statistical analysis was by two-way ANOVA or mixed-effects analysis with Tukey’s multiple comparisons test. The letters (“a”, “b”, “ab” etc) denote the outcome of statistical comparisons with statistically significant differences (*p* < 0.05) indicated by discrepant letters, whereas identical letters indicate *p* > 0.05. All exact *p* values are provided in Supplementary Data 1. **d** Quantification of sinusoidal trophoblast giant cells (sTGCs) in WT, HET, and KO placentas of the three lines. Data are displayed as mean ± SEM from *Atp11a*: WT *n* = 8, HET *n* = 9, KO *n* = 7; *Smg9*: WT *n* = 8, HET *n* = 8, KO *n* = 7; *Ssr2*: WT *n* = 9, HET *n* = 6, KO *n* = 7 placentas. Significance was evaluated by mixed-effects analysis with Tukey’s multiple comparisons test. **e** Immunofluorescence staining for MCT1 (SLC16A1) demarcating the syncytiotrophoblast-I layer of the placental labyrinth, indicative of the overall extent of placental vascularization. **f** Higher magnification views of the placental labyrinth zone stained for MCT1 (red) and MCT4 (SLC16A3, green), highlighting the dilated appearance of maternal blood sinusoids and fetal vessels, respectively. Images in **e**, **f** are representative of sample numbers detailed below. **g** Quantification of fetal and maternal blood spaces as determined by MCT4- and MCT1-enclosed areas, respectively. **h** MCT1- and MCT4-defined perimeters of the syncytial layers facing maternal blood spaces and fetal blood vessels. Data in **e**–**h** are from three representative labyrinthine areas of *Atp11a*: WT *n* = 8, HET *n* = 9, KO *n* = 7; *Smg9*: WT *n* = 8, HET *n* = 8, KO *n* = 7; *Ssr2*: WT *n* = 8, HET *n* = 6, KO *n* = 8 placentas displayed as mean ± SEM. Statistical analysis was by mixed-effects analysis with Tukey’s multiple comparisons test. Source data are provided as a Source Data file. All exact *p* values are provided in Supplementary Data 1.

In parallel to the embryos, one-half of their corresponding placentas were analyzed in detail for various histological parameters, including cross-sectional area (by H&E staining, *Tpbpa* in situ hybridization), the relative contributions of the main placental layers (by *Tpbpa* in situ hybridization), the number of sinusoidal trophoblast giant cells (=sTGCs, by RXRa and DAPI staining), and the structure of the maternal and fetal vascular spaces in the labyrinth (by MCT1/MCT4 staining) (Fig. 2a–h). Overall, mutant placentas of all three lines were about one-fifth to one-third smaller compared to WT, with the *Smg9*^−/−^ placentas being most severely affected (Fig. 2b, c and Supplementary Fig. 2a). Although the junctional zone of *Smg9* mutants was disproportionately smaller, the overall placental size reduction was primarily due to a proportional decrease of all three principal layers–decidua, junctional zone, and labyrinth (Fig. 2c). The number of sTGCs, a layer of hormone-producing cells in the placental labyrinth that are reduced in *Pparg* mutants, trended upwards in *Atp11a*^−/−^ placentas but was significantly decreased in *Smg9*^−/−^ and *Ssr2*^−/−^ placentas (Fig. 2d). The labyrinthine architecture was disrupted in all three lines (Fig. 2e, f), with abnormally dilated fetal and/or maternal vascular areas, indicative of reduced branching morphogenesis (Fig. 2e–h). Moreover, the perimeters of fetal and maternal blood spaces were markedly reduced in *Atp11a*^−/−^ and *Ssr2*^−/−^ placentas, a measure of a smaller feto-maternal transport surface area (Fig. 2h). Overall, these defects point to vascular insufficiencies of mutant placentas in all three lines.

Consistent with the finding that *Smg9*^−/−^ placentas exhibited the most severe overall size phenotype, principal component analysis (PCA) of placental transcriptomes clearly distinguished the *Smg9* mutant samples from WT and HET placentas (Supplementary Fig. 2b). *Atp11a*-null placentas partially separated from WT and HET controls along principal component (PC) 2, whereas the three genotypes of the *Ssr2* samples did not display a clear-cut clustering based on global expression profiles (Supplementary Fig. 2b). Gene enrichment term analysis using WebGestalt^37^ and other gene ontology (GO) search algorithms (Metascape, EnrichR, etc) on differentially expressed (DE) genes for each mutant line revealed themes related to reproduction, cell growth and proliferation, cell-cell adhesion, and chemokine and hormone signaling pathways (Supplementary Fig. 2b). As expected from their pronounced transcriptomic divergence, these terms reached highest significance for the *Smg9*^−/−^ placentas. Most DE genes were unique to each of the mutant lines, with only a modest overlap between the three (Supplementary Fig. 2c). The most relevant GO terms shared between all three lines related to the regulation of cell morphogenesis and pregnancy (i.e., lactation) (Supplementary Fig. 2d).

### Trophoblast-specific cKO generation

**Fig. 3:**
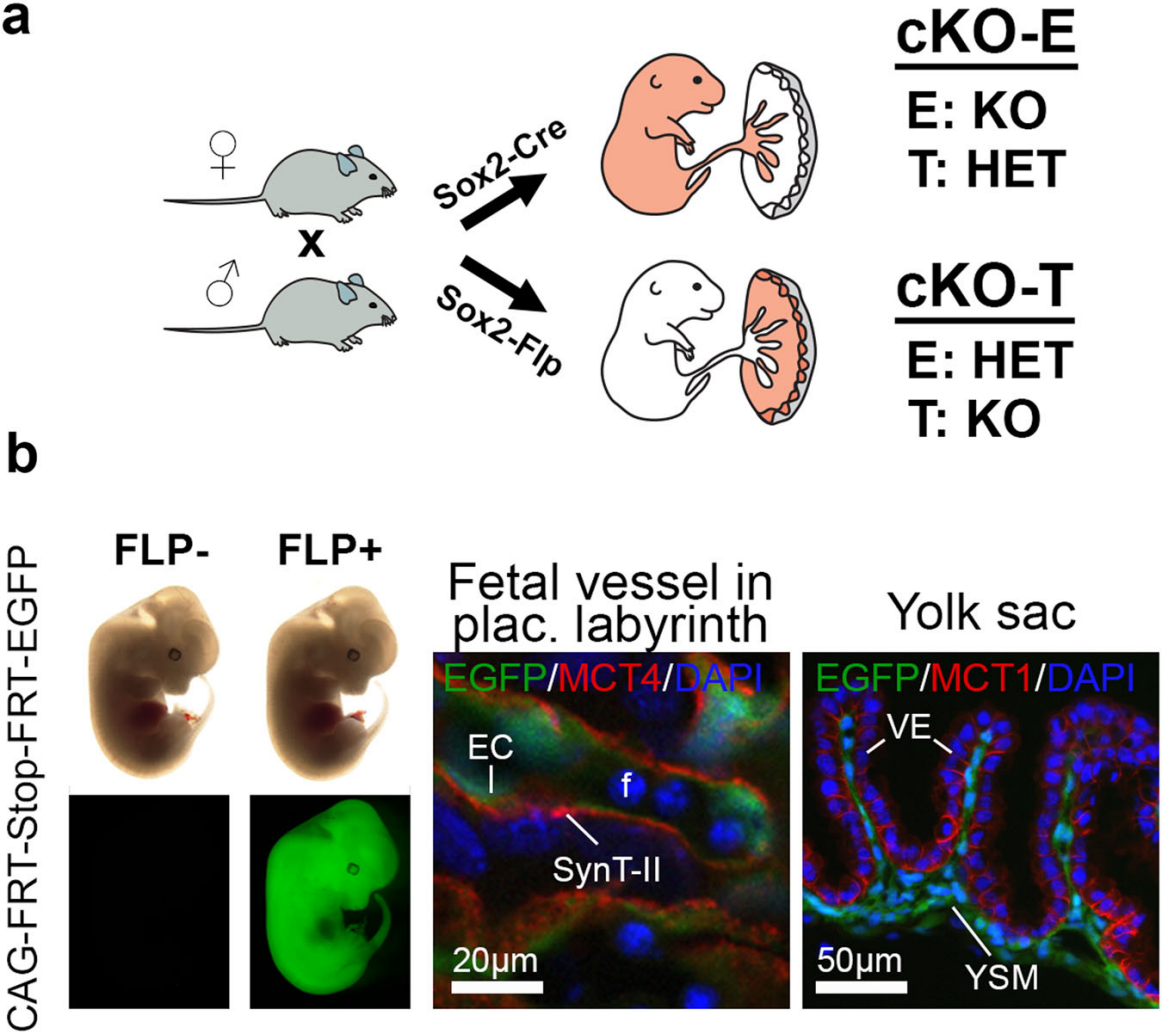
Conditional knockout strategies to determine causality of pathologies. **a** Outline of *Sox2*-Cre and *Sox2*-Flp recombinase driver strategy to generate embryo-specific (cKO-E) and trophoblast-specific (cKO-T) knockouts (see also Supplementary Fig. 3). E embryo, T placental trophoblast. The rendering of the embryo-placenta schematic is from Perez-Garcia, et al. Placentation defects are highly prevalent in embryonic lethal mouse mutants. Nature 555, 463-468 (2018). **b** Cell lineage-specific activity of the *Sox2*-Flp transgenic line, as established by mating of *Sox2*-Flp males to females carrying a FLP-activatable enhanced green fluorescent protein (EGFP) reporter transgene. EGFP expression is observed in the entire embryo of FLP-positive (FLP+) but not FLP-negative (FLP−) E12.5 embryos. In extra-embryonic tissues, EGFP is present in endothelial cells (EC) of fetal blood vessels (f) of the placental labyrinth, and in yolk sac mesoderm (YSM). These EGFP-positive cell types are of epiblast lineage origin. MCT4-demarcated SynT-II cells and MCT1-positive visceral endoderm (VE) are EGFP-negative, as expected.

The ultimate goal of this study was to determine placental trophoblast- versus embryonic lineage-specific contributions to the heart phenotype. Here, we took advantage of the tm1a allele structure of the KOMP/EUCOMM alleles^26^ that allows conversion either into a constitutive KO tm1b allele by CRE recombinase, or restoration of the KO-first allele to a functional, floxed tm1c allele by FLP recombinase action (Supplementary Fig. 3a). Using floxed alleles in combination with paternally inherited *Sox2*-Cre^25,38^ is a common strategy for generating embryo-specific cKO (cKO-E) conceptuses (Fig. 3a and Supplementary Fig. 3b). However, this genetic constellation is insufficient to dissect the net impact of placental trophoblast on phenotype severity. Unambiguous proof that the placenta causes CHD requires the generation of a trophoblast-specific cKO (cKO-T).

To facilitate the generation of trophoblast-specific gene deletion by genetic means without the need for extensive embryo manipulation, we established a new transgenic line, *Sox2*-Flp, which expresses optimized FLP recombinase (FlpO) under the control of the same epiblast-specific *Sox2* promoter-enhancer construct originally used for the *Sox2*-Cre mouse line^25^. We assessed this new transgenic line for cell type-specific FLP activity using an FLP-activated EGFP reporter line. This approach demonstrated EGFP activation in the E5.5 epiblast but not in trophoblast cells of the extra-embryonic ectoderm (Supplementary Fig. 3c). In line with this activity pattern, at E12.5 EGFP was detected in the entire embryo in FLP-positive (FLP+) but not FLP-negative (FLP-) conceptuses (Fig. 3b). Moreover, EGFP expression was present in fetal endothelial cells of the placental labyrinth as well as in yolk sac mesoderm, as expected as these cell types are derived from extra-embryonic mesoderm of epiblast origin (Fig. 3b and Supplementary Fig. 3d). By contrast, cells of the trophoblast lineage remained EGFP-negative, specifically the MCT1- and MCT4-demarcated syncytiotrophoblast cells of the placental labyrinth (Fig. 3b and Supplementary Fig. 3d). As expected, junctional zone trophoblast also lacked EGFP expression (Supplementary Fig. 3d), with the exception of one placenta in which <1% of junctional zone trophoblast cells were EGFP+. To further verify full *Sox2*-Flp functionality in the embryo, we also used the *Ssr2* tm1a allele as a reporter and demonstrated complete, non-mosaic FLP-mediated tm1a->tm1c allele conversion throughout the entire embryo proper as well as in the fetal vasculature of the placenta (Supplementary Fig. 3e, f). To ascertain the retention of the tm1a KO allele in the junctional zone specifically in the *Ssr2* line where this gene is expressed (Supplementary Fig. 1c), we performed whole-mount LacZ stainings on bisected placentas and confirmed that the junctional zone was left unaffected by *Sox2*-Flp action (Supplementary Fig. 3f). Finally, we performed extensive genotyping of fine-dissected E8.5 conceptuses to verify complete tm1a->tm1c allele conversion in the embryo and allantoic (extra-embryonic) mesoderm, retention of tm1a in trophoblast tissues, and a mixed allele composition in yolk sac that consists of mesoderm and primitive endoderm derivatives, as expected (Supplementary Fig. 4). These comprehensive verification assays proved that our new *Sox2*-Flp transgenic line was a powerful tool to revert KO-first alleles in the entire embryo proper while leaving trophoblast largely unaffected, thus enabling a streamlined genetics-based generation of cKO-T conceptuses from existing mouse KO resources^26^.

### Dissecting lineage-specific gene function

Applying the *Sox2*-Cre and *Sox2*-Flp drivers to generate cKO-E and cKO-T conceptuses, respectively, enabled us to genetically differentiate between embryo-specific and trophoblast-specific contributions of gene function to the heart phenotypes in each of the three mouse mutant lines (Fig. 3a). For direct comparisons, the phenotypic analyses of cKO embryos and placentas followed the same procedures used earlier with the constitutive KOs.

**Fig. 4:**
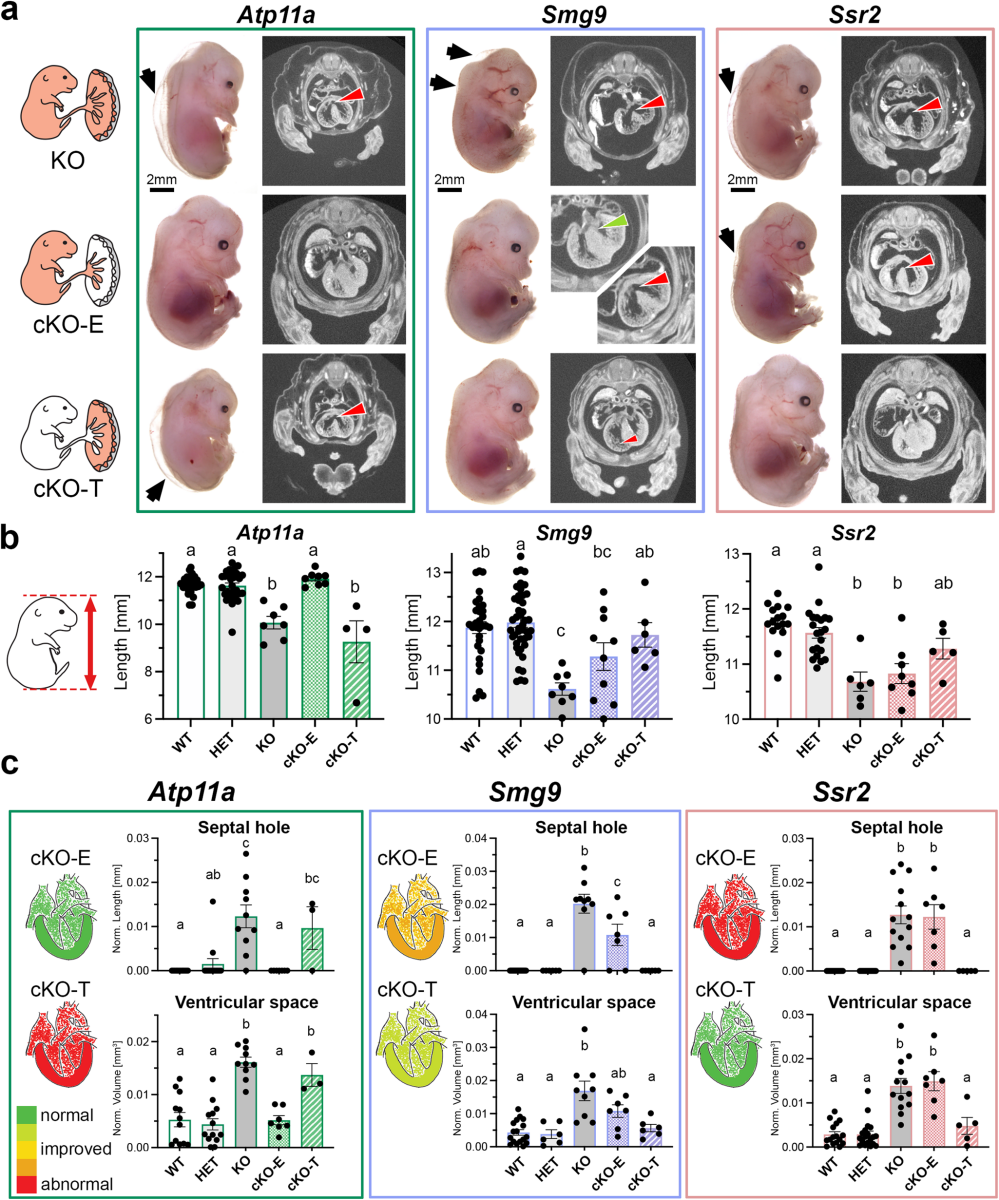
Conditional knockout analysis of selected mouse mutant strains. **a** Representative photos of embryos and coronal µCT sections through the heart summarizing the outcomes of the conditional knockout strategies. Black arrows highlight edema, red arrowheads ventricular septal defects in the heart. Edema is also evident in the µCT scans by the gap between the skin and the solid tissue layer. For *Smg9* cKO-E, the green arrow highlights a fully closed and well-developed ventricular septum, in contrast to constitutive KOs. *Smg9* cKO-T hearts are in general developmentally improved compared to the constitutive KO. The rendering of the embryo-placenta schematic is from Perez-Garcia, et al. Placentation defects are highly prevalent in embryonic lethal mouse mutants. Nature 555, 463-468 (2018). **b** Embryo crown-rump length measurements of all 5 genotypes per strain. Data are displayed as mean ± SEM of *Atp11a*: WT *n* = 23, HET *n* = 30, KO *n* = 7, cKO-E *n* = 8, c-KO-T *n* = 4; *Smg9*: WT *n* = 31, HET *n* = 44, KO *n* = 8, cKO-E *n* = 10, c-KO-T *n* = 6; *Ssr2*: WT *n* = 16, HET *n* = 21, KO *n* = 6, cKO-E *n* = 8, c-KO-T *n* = 5 embryos. Statistical analysis was performed using one-way ANOVA with Tukey’s multiple comparisons test. The letters indicate statistical comparisons, where identical letters between samples mean no difference, but discrepant letters indicate significant changes. **c** Assessment of heart phenotypes in cKO embryos of all genotypes, displaying the two most informative measurements—ventricular hole size and ventricular septal space as inverse measure of myocardial thinning. Data are displayed as mean ± SEM. Statistical analysis was performed using one-way ANOVA with Holm–Šídák’s multiple comparisons test. Statistically significant differences (*p* < 0.05) are indicated by distinct letters, whereas same letters represent *p* > 0.05. Schematics of hearts are color-coded to display the degree of abnormality in the various genetic constellations. Measurements were taken from individual control (WT/HET) and KO (KO, cKO) embryos matched across litters for *Atp11a* (WT *n* = 12, HET *n* = 13, KO *n* = 10, cKO-E *n* = 7, c-KO-T *n* = 3), *Smg9* (WT *n* = 17, HET *n* = 6, KO *n* = 9, cKO-E *n* = 7, c-KO-T *n* = 6), *Ssr2* (WT *n* = 16, HET *n* = 19, KO *n* = 13, cKO-E *n* = 7, c-KO-T *n* = 5). Source data are provided as a Source Data file. All exact *p*-values are provided in Supplementary Data 1.

For *Atp11a*, 7/8 (87.5%) of the cKO-E embryos appeared grossly normal (Fig. 4a, b and Supplementary Fig. 5a, b), with only one embryo exhibiting mild edema. In line with this observation, none of the hearts of normal-appearing cKO-E embryos showed signs of myocardial thinning or VSDs, in stark contrast to the constitutive KOs (Fig. 4a–c and Supplementary Fig. 5a). Placental morphology of cKO-E conceptuses was also largely normal, including the labyrinth vasculature that was profoundly affected in the constitutive KO (Supplementary Fig. 5c, d). cKO-E embryos survived throughout gestation and live, healthy, and fertile *Atp11a*^−/−^ animals were obtained (Supplementary Fig. 5e). These data demonstrate that ATP11A is not required in the embryonic lineage for normal development.

Most importantly, we also assessed *Atp11a* cKO-T conceptuses in which any embryonic phenotype is solely driven by placental trophoblast defects. *Atp11a* cKO-T embryos were disproportionally absent by E14.5, strongly suggesting that many succumbed earlier during development (Supplementary Fig. 5f, g); a total of four E14.5 cKO-T embryos and two additional E16.5 cKO-T embryos that survived early attrition were recovered. All four E14.5 cKO-T embryos were severely affected, with one too necrotic for analysis by µCT imaging, as were both of the cKO-E embryos recovered at E16.5 (Fig. 4a and Supplementary Fig. 5h). Defects in the remaining three E14.5 embryos were as profound as in the constitutive KOs (Fig. 4a–c and Supplementary Fig. 5a, b, d). They were extremely edematous, developmentally delayed, and moribund, with severe thinning of the myocardial wall and a profound VSD in two of the three, as in the constitutive KO (Fig. 4a–c and Supplementary Fig. 5a). These data unequivocally show that *Atp11a* gene function is required exclusively in the trophoblast lineage of the placenta for normal cardiogenesis and overall embryonic development and viability.

The apparent exacerbation of phenotypes in the *Atp11a* cKO-T constellation may be explained by the allele structure of these embryos; constitutive KOs are genetically made up of two tm1a alleles that allow sporadic splicing events across the knockout cassette, which we find can produce average ~10% functional transcript per allele for some targeted loci (Supplementary Fig. 6a). By contrast, cKO-T placentas carry one tm1a allele and one tm1b allele. The latter is deleted for the targeted exon, thus precluding any possibility of a functional transcript being produced (Supplementary Fig. 3a, b). This discrepancy leaves room for some quantitative variation in phenotypes for genes that are acutely sensitive to exact gene dosage.

Conditional ablation of *Smg9* in the embryo or placental trophoblast resulted in substantial inter-individual variation. cKO-E embryos were identifiable on dissection by a smaller size, edematous and/or paler appearance, but overall were not as severely affected as the constitutive KO (Fig. 4a–c). This partial rescue was reflected in intermediate crown-rump length and heart phenotypes of cKO-E embryos that positioned them in between the WT and constitutive KO, in particular for septal hole size (Fig. 4a–c and Supplementary Fig. 5a). Two cKO-E embryos presented with no VSD at all, indicating that *Smg9* function in trophoblast plays a role in heart development. In the reciprocal cKO-T configuration, three embryos appeared overtly normal, whereas another three were edematous and/or smaller. These divergent phenotypes were unrelated to fetal sex (Supplementary Fig. 6b). Heart analyses did not detect a VSD or a statistically significant difference in myocardial wall thickness, although some embryos clearly exhibited a thinning of the trabecular layer, in particular of the right ventricle, and reduced compact layer thickness (Fig. 4a and Supplementary Figs. 5a, 6c). The placental size reduction observed in the constitutive *Smg9* KO was significantly improved in both, cKO-E and cKO-T conceptuses, and the vascular organization of the labyrinth compartment was similar to WT (Supplementary Fig. 5c, d). These findings suggest an additive or synergistic effect of *Smg9* deletion in the embryonic and trophoblast lineages. This crosstalk plays out most notably in the placenta where both lineages are in close juxtaposition to each other. Importantly, the penetrance and expressivity of the *Smg9*^−/−^ heart phenotype cannot be explained by gene function in the embryo alone, indicating a critical contribution by placental trophoblast.

Finally, for *Ssr2*, 7/8 (87.5%) cKO-E embryos exhibited edema, developmental delay, and sometimes additional defects such as severe craniofacial and ocular abnormalities (Fig. 4a). Concerning heart pathologies, *Ssr2* cKO-E embryos exhibited VSDs and myocardial thinning, just as the constitutive KOs (Fig. 4a–c; Supplementary Fig. 5a). Conversely, the cKO-T embryos appeared largely normal, although their placentas remained somewhat smaller (Fig. 4a–c and Supplementary Fig. 5c, d). These data show that the origin of the heart defects in *Ssr2* mutants is predominantly intrinsic to the embryonic lineage with no or only minor contributions by the placental trophoblast compartment. Accordingly, and in keeping with their normal phenotypic appearance, *Ssr2* cKO-T embryos were viable and survived into adulthood.

### Atp11a-specific placental defects

**Fig. 5:**
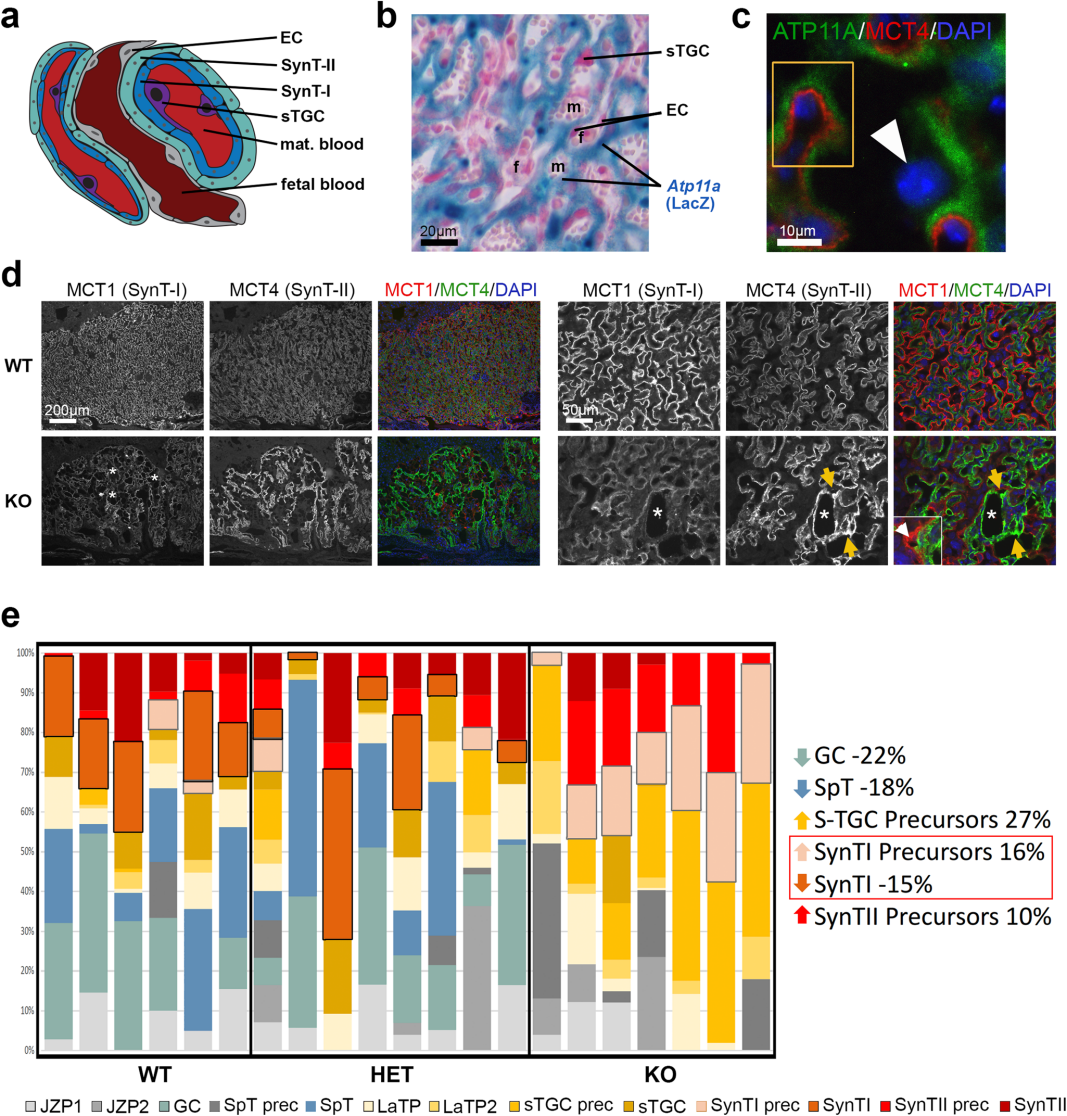
Specific placental pathologies in *Atp11a* mutants. **a** Diagram of the fine-structural organization of the mouse placental labyrinth, which forms the feto-maternal exchange area. EC = endothelial cells; SynT-I = syncytiotrophoblast layer I; SynT-II = syncytiotrophoblast layer II; sTGC = sinusoidal trophoblast giant cell; mat. blood = maternal blood. **b** LacZ staining of an *Atp11a*^+/−^ placenta in which LacZ serves as readout of endogenous *Atp11a* expression. *Atp11a* expression locates to the syncytiotrophoblast, whereas endothelial cells (EC) lining fetal blood (f) vessels and sinusoidal trophoblast giant cells (sTGCs) facing maternal blood (m) sinusoids are not stained. Staining is representative of *n* = 6 placentas. **c** Double immunofluorescence staining for beta-galactosidase as indicator of ATP11A expression (green) and for MCT4 (red) as marker of the syncytiotrophoblast layer-II (SynT-II). The closely juxtaposed but non-overlapping staining (yellow rectangle) demonstrates that ATP11A is confined to SynT-I. The arrowhead points to an sTGC negative for ATP11A. Stainings are representative of *n* = 3 placentas. **d** MCT1/MCT4 double immunofluorescence staining on *Atp11a* WT and KO placentas, imaged at identical exposure setting. Asterisks demarcate areas of severe vascular disruptions. MCT4 staining intensity is increased, but MCT1 staining decreased in *Atp11a* mutant placentas. The SynT-II layer labeled by MCT4 is also increased in thickness, often delaminated from the SynT-I layer (inset) and exhibits pathological accumulations (yellow arrows). Images are representative of *n* ≥ 3 samples per genotype. **e** Virtual deconvolution of placental RNA-seq data for cell type-specific composition^39,40^ reveals an under-representation of mature SynT-I, glycogen cells (GCs) and spongiotrophoblast (SpT) cells, but an enrichment of precursors (prec) of SynT-I, SynT-II, and GCs in *Atp11a*-mutant placentas. Each bar represents an individual placenta, with absolute values of the proportional cell type composition being depicted. Statistically significant cell type differences are given on the right; comparisons were made with a two-tailed Student’s *t* test. All *p* values are provided in Supplementary Data 1. JZP = junctional zone precursor, LaTP = Labyrinth precursor (WT *n* = 6, HET *n* = 8, KO *n* = 7). Source data are provided as a Source Data file.

Given that *Atp11a* provided a clear-cut example of a heart phenotype that is exclusively driven by gene function in trophoblast, we examined the placental abnormalities of this mouse line in greater depth. Recent single nuclei-RNA sequencing (snRNA-seq) data identified *Atp11a* as a marker of the SynT-I layer in the murine placental labyrinth^39^, i.e., the cells directly facing maternal blood (Fig. 5a). Based on LacZ reporter gene expression coupled with immunostaining for the two syncytiotrophoblast layers, we confirmed that *Atp11a* expression was indeed confined to the SynT-I cell layer, with no obvious expression in SynT-II, sTGCs or endothelial cells of the fetal vasculature (Fig. 5b, c and Supplementary Fig. 7a, b). Double immunofluorescence staining for MCT1, encoded by the *Slc16a1* gene and a marker of SynT-I, and MCT4 (*Slc16a3*) demarcating the SynT-II layer, revealed major structural abnormalities in *Atp11a*^−/−^ placentas. Specifically, mutant placentas lacked the intricate, finely branched organization of the labyrinthine architecture, and the tight apposition of the SynT-I and -II layers was frequently disrupted (Fig. 5d, inset). Many areas appeared acellular with large holes in the vascular structure. In more severely affected or moribund cKO-T conceptuses, these malformations of the labyrinthine architecture were even further exacerbated with a complete spatial dissociation of the two syncytial layers (Supplementary Fig. 7c). Moreover, it was obvious that under identical experimental conditions, MCT1 staining was significantly weaker and the MCT4 signal markedly stronger in KO compared to WT placentas (Fig. 5d). The SynT-II layer also contained accumulations of MCT4 staining at sinusoidal branch points, indicative of a thickened, disorganized interhaemal barrier.

To investigate the trophoblast defect of *Atp11a* mutant placentas more closely, we applied the BisqueRNA package to our placental RNA-seq data, a bioinformatic method designed to estimate cell composition from bulk expression data^40^. This analysis revealed that *Atp11a*^−/−^ placentas were significantly deficient in mature SynT-I cells, whereas markers of precursors of SynT-I and SynT-II were enriched (Fig. 5e and Supplementary Fig. 7d). These data imply that both syncytial layers fail to undergo normal differentiation in the absence of *Atp11a*, with the SynT-I layer being more severely affected. Although mature spongiotrophoblast and glycogen cells were also under-represented in this transcriptional deconvolution analysis, these deficits were less obvious histologically (Fig. 2).

### Placental CHD models share SynT-I defect

**Fig. 6:**
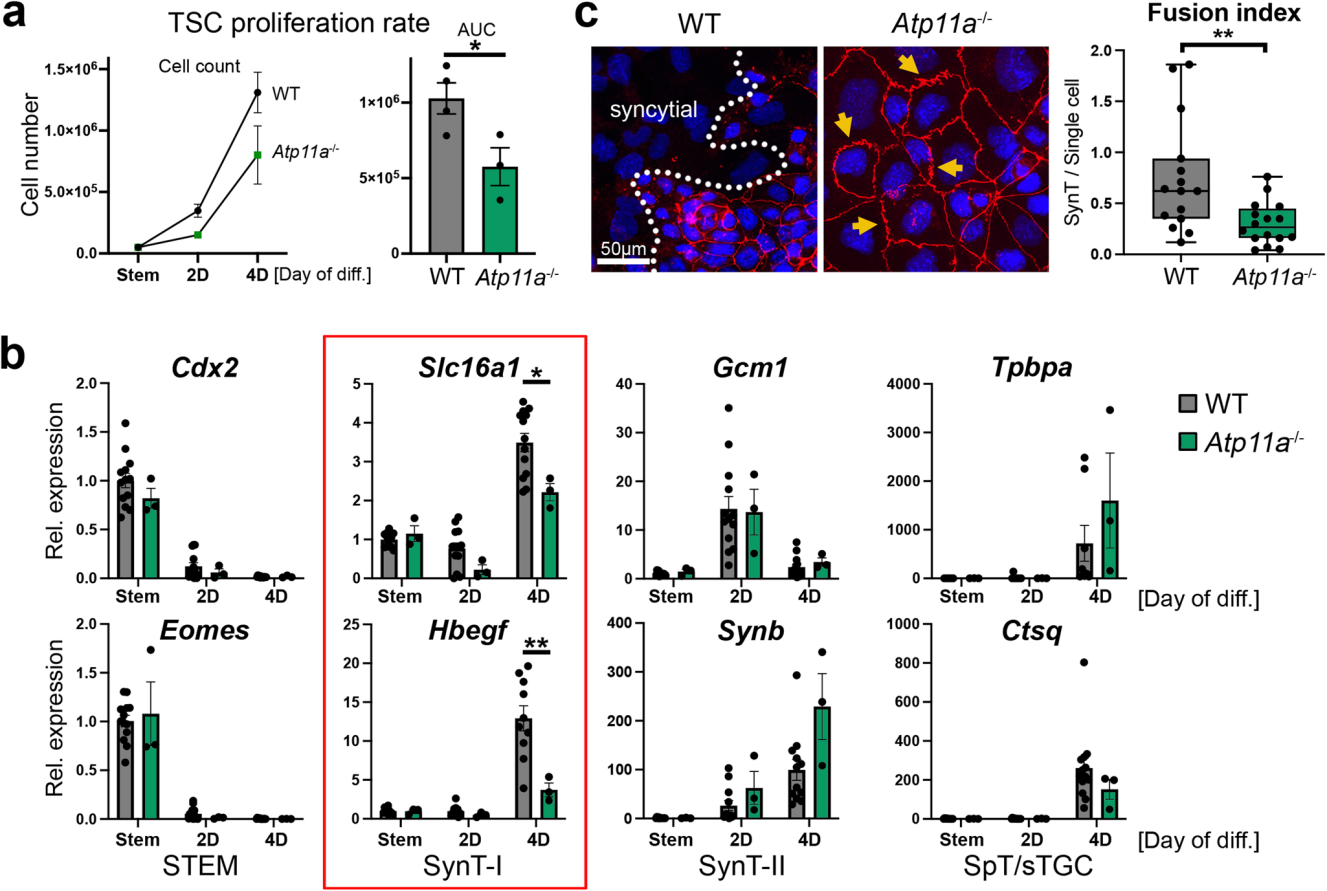
*Atp11a*-mutant TSCs display SynT-I defects. **a** Proliferation rates of WT and *Atp11a*^−/−^ trophoblast stem cells (TSCs) over a 4-day differentiation time course. AUC = area under curve. Data are from individual WT (*n* = 4) and KO (*n* = 3) clones each assessed in 3 independent replicate experiments, displayed as mean ± SEM. Two-tailed Student’s *t* test, * *p* < 0.05. **b** RT-qPCR analysis of cell type-specific marker genes on WT (*n* = 11) and *Atp11a*^−/−^ KO (*n* = 3) TSCs during a 4-day differentiation time course. *Atp11a*^−/−^ TSCs exhibit a SynT-I differentiation defect. Data are normalized to WT cells in stem cell conditions (Stem) and displayed as mean ±SEM (two-way ANOVA, Tukey’s multiple comparisons test, * *p* < 0.05, ** *p* < 0.01). **c** Assessment of cell fusion capacity of WT and *Atp11a*^−/−^ TSCs upon 4 days of differentiation. ZO-1 cell membrane staining (red) highlights membrane ruffling (arrows) and fewer syncytialised areas in *Atp11a*^−/−^ TSCs. Data are from WT: *n* = 15 and *Atp11a*^−/−^: *n* = 16 images of two independent clones per genotype amounting to >1600 counted nuclei each. The box plot shows the 25th to 75th percentiles with the median indicated, and the whiskers stretch from the minimum to the maximum values, displaying all data points (Two-tailed Student’s *t* test, ** *p* < 0.01). Source data are provided as a Source Data file. All exact *p* values are provided in Supplementary Data 1.

In the context of the whole placenta, syncytiotrophoblast formation is influenced by extrinsic factors such as the obligate interaction with fetal endothelial cells and maternal blood flow. To tease out the trophoblast-intrinsic defects induced by lack of ATP11a, we established *Atp11a*-mutant trophoblast stem cells (TSCs) by CRISPR/Cas9 technology. *Atp11a* null TSCs were morphologically indistinguishable from their WT counterparts when cultured in stem cell conditions. However, upon induction of differentiation, their proliferation rates dropped significantly faster compared to WT TSC clones (Fig. 6a). Differentiation time course experiments coupled with the analysis of trophoblast cell type-specific marker gene expression revealed a specific defect in SynT-I formation (Fig. 6b and Supplementary Figs. 8, 9). This deficit was most robustly detected on day 4 of TSC differentiation when syncytiotrophoblast differentiation peaked. By contrast, SynT-II cells were not markedly affected even in directed differentiation experiments (Supplementary Fig. 8b), and neither could we detect a difference in differentiation rates of sTGCs or other trophoblast cell types in extended TSC differentiation experiments (Supplementary Fig. 9). We also scored the fusion capacity of *Atp11a*-null TSCs compared to WT, and found a drastic decrease in syncytium formation after 4 days of differentiation (Fig. 6c). Instead of forming syncytia, *Atp11a*^−/−^ cells often displayed ruffled cell membranes that failed to break down for cell fusion (Fig. 6c). Overall, these in vivo and in vitro data demonstrate that *Atp11a* deletion interferes predominantly with SynT-I differentiation.

**Fig. 7:**
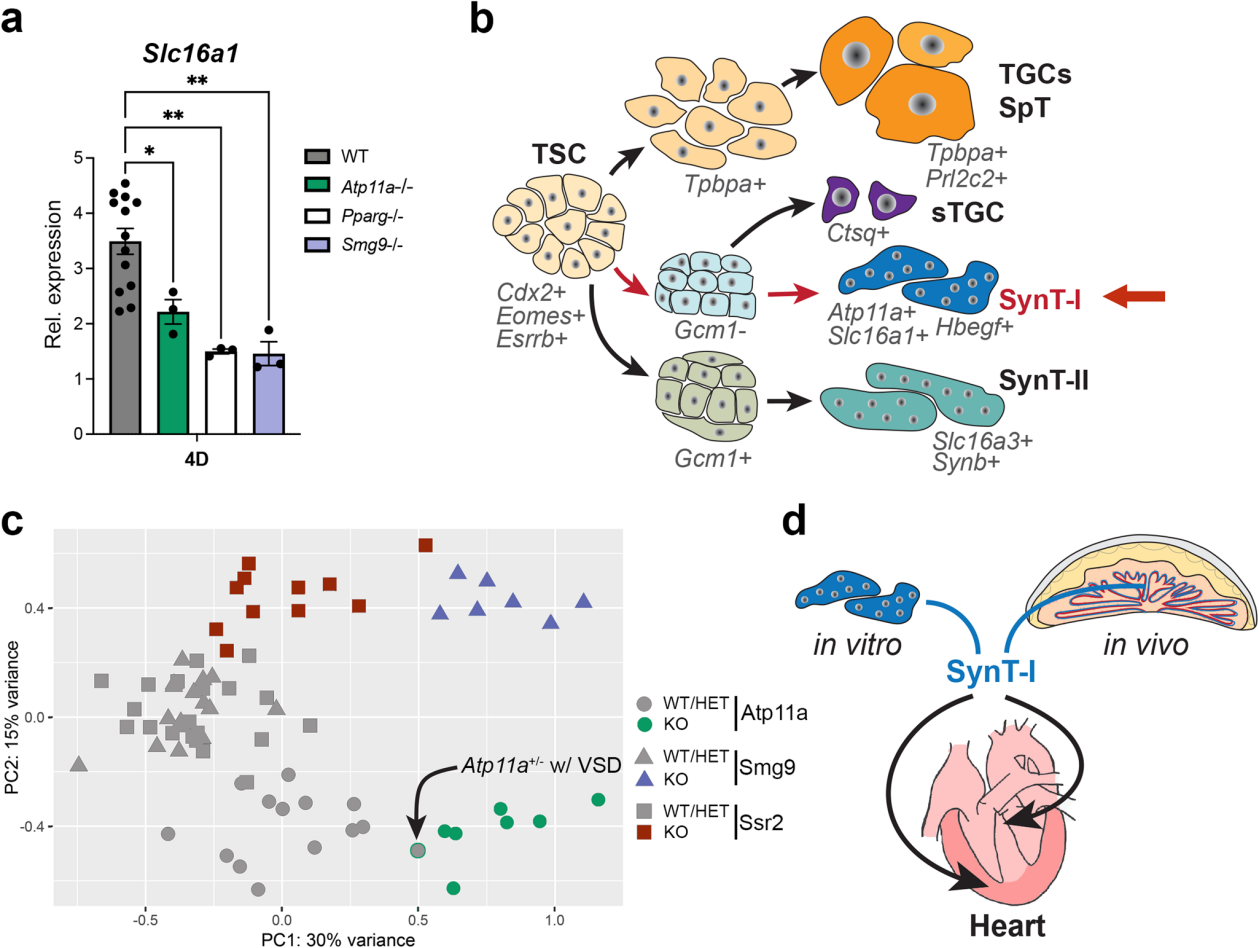
SynT-I defects correlate with cardiac pathologies. **a** RT-qPCR analysis of SynT-I marker *Slc16a1* (=MCT1) in WT (*n* = 11), *Atp11a*- (*n* = 3), *Pparg*- (*n* = 3) and *Smg9*-mutant (*n* = 3) TSC clones after 4 days of differentiation. Data are from independent clones each, displayed as mean ± SEM (one-way ANOVA, Holm-Šídák’s multiple comparisons test,* *p* < 0.05, ** *p* < 0.01). **b** Schematic diagram of major TSC differentiation trajectories, highlighting the SynT-I pathway compromised in *Atp11a*^−/−^, *Pparg*^−/−^, and *Smg9*^−/−^ TSCs. **c** Principal component analysis of bulk placental RNA-seq data filtered for SynT-I marker genes^39^. SynT-I expression patterns separate all KO samples (*n* = 25) from unaffected WT and HET samples (*n* = 51). The highlighted datapoint is an *Atp11a*^+/−^ placenta of an embryo exhibiting VSD; it clusters closer to the KO samples (Fig. 1f). **d** Diagram depicting the apparent connection between SynT-I differentiation and heart development. Source data are provided as a Source Data file. All exact *p*-values are provided in Supplementary Data 1.

*Pparg* was the first mouse mutation in which a placental cause of a heart defect was demonstrated. This was accomplished by using tetraploid aggregation experiments which supply the mutant embryo with functional trophoblast cells, akin to the *Sox2*-Cre generated cKO-E conceptuses^11^. With this in mind, we wanted to extend our findings from the *Atp11a* mutants to explore whether defective syncytiotrophoblast differentiation was at the core of placentally-induced heart defects in the *Pparg* paradigm model as well. For this reason, we established *Pparg*^−/−^ TSCs by CRISPR/Cas9-mediated deletion; we also generated *Smg9*^−/−^ TSCs to investigate whether the milder trophoblast effect on heart development in this mouse line was still associated with SynT-I defects. Indeed, we found that SynT-I differentiation deficiencies were the common denominator in all these mutant TSC lines, with lower expression of the monocarboxylate transporter *Slc16a1* (MCT1) and the heparin-binding EGF-like growth factor *Hbegf* consistently providing the most reliable readout (Fig. 7a, b and Supplementary Figs. 8, 9). By contrast, other classical SynT-I markers, notably *Syna* as well as *Bmper*^39^, were not consistently down-regulated. To investigate this phenomenon further, we reclustered previously published snRNA-seq^39^ data specifically on syncytiotrophoblast cells, and found that *Atp11a*, *Slc16a1* (MCT1), *Hbegf,* and *Pparg* were enriched in two particular sub-clusters demarcating specific subsets of SynT-I nuclei (Supplementary Fig. 10a). These data provide an intriguing explanation for the common de-regulation of these genes in our mutant TSCs.

### SynT-I defects are indicative of CHD

Faulty SynT-I differentiation can be caused by problems intrinsic to the trophoblast progenitor cell population itself, or by aberrant inductive signals of adjacent endothelial and SynT-II cells^41–43^. Based on our insights, it is, therefore, possible that abnormal SynT-I expression profiles may be indicative of heart defects regardless of whether they arise as a result of primary gene function in this cell type (*Atp11a*, *Smg9*, *Pparg*) or because they are extrinsically induced (*Ssr2*). This rationale prompted us to investigate SynT-I differentiation in all our mutant placentas. BisqueRNA analysis indeed detected a SynT-I differentiation defect in all our mutant placentas, which was most significant in the *Atp11a*- and *Smg9*-null placentas (Supplementary Fig. 7d). Moreover, by applying the SynT-I signature gene set based on recent single cell analyses^39^ to our bulk RNA-seq data, mutant samples of all three lines (*Atp11a*, *Smg9*, *Ssr2*) were distinctly separated from their unaffected WT and HET counterparts (Fig. 7c). This clustering was far more clear-cut than that based on global gene expression profiles, which did not fully separate KO samples from their WT and HET counterparts in the *Atp11a* and *Ssr2* lines (Supplementary Fig. 2). SynT-I-directed clustering even identified the *Atp11a*^+/−^ conceptus that exhibited a mild VSD (Fig. 1f and Fig. 7c). By contrast, SynT-II-, glycogen cell-, spongiotrophoblast- and sTGC-based clustering did not identify the mutant samples as clearly (Supplementary Fig. 10b). These data indicate that placental gene expression patterns, specifically those tied to SynT-I, correlate with embryonic heart phenotypes irrespective of the developmental origin of the CHD. SynT-I differentiation defects may therefore link placental development and function to deficiencies in cardiogenesis (Fig. 7d).

## Discussion

The existence of a ‘placenta-heart axis’ has been identified over 20 years ago. Despite that, in most studies the placenta is not considered as a possible cause or contributor to developmental heart defects. Over 3,500 individual gene mutations are associated with a heart phenotype in the mouse (Mouse Genome Informatics), but only very few KO studies have tested for a potential placental contribution to the embryonic phenotype^8,11–17^. A systematic exploration aimed at gaining an estimate of the global impact of the placenta on heart development is missing. Here, we undertook an unbiased approach by selecting three mouse mutant lines primarily on the basis of exhibiting placenta and heart phenotypes. Even though the number of assessed genes remains small due to the massive undertaking of this genetic approach, our study reveals that the placenta is fully causative of heart defects and of embryonic lethality in one out of three mouse mutant lines, and partially contributes to the heart phenotype in a second line. In support of our findings, a very recent study also reported the rescue of intrauterine lethality and heart defects in *Atp11a* embryo-specific KOs^44^. In the case of *Smg9*, our data reveal the importance of additive or synergistic gene function in both the trophoblast and embryonic lineage compartments. Overall, these findings suggest a prominent role of the placenta in the etiology of CHD. In humans, a correlation between placental abnormalities and CHD has been reported^45–47^, but cause-effect relationships cannot be determined. However, with over 50% of human CHDs remaining classified as idiopathic^3–6^, the placenta may well represent the missing etiological link in many of these unexplained cases.

The principal heart defects observed in this study were VSDs, coupled with outflow tract alignment defects and ventricular wall thinning. VSDs are the most common subtype of CHDs in humans that can have several underlying causes. They can occur as an isolated defect related to the fusion of the conotruncal septum with the interventricular septum. However, the occurrence of outflow tract alignment defects strongly suggests a primary deficiency in the contribution of second heart field progenitors^48^. Alternatively, the thinness of the myocardium in mutant embryos suggests a deficiency in cardiomyocyte proliferation, which can also cause a VSD by inadequate growth of the muscular portion of the interventricular septum.

The mouse provides a powerful experimental model to test for phenotype causality using tetraploid aggregation assays^49^ or lineage-specific Cre drivers^25,50^. In the few instances where this has been applied, the most common constellation is a rescue of the placental trophoblast while leaving the embryo deficient for the gene of interest. In this scenario, if the heart defect and the associated embryonic lethality are improved or rescued, it can be reasoned that the origin of the phenotype resides in the placenta. However, these strategies cannot deduce the net impact of trophoblast defects on the heart. Here, we establish a new transgenic mouse, *Sox2*-Flp, that overcomes these issues. This new genetic tool can be used in conjunction with the broad collection of publicly available KOMP/EUCOMM-targeted alleles to generate trophoblast-specific KOs. This powerful methodological advance will help elucidate the contribution of the placenta to embryonic phenotypes and to long-term adult health and disease predisposition on a genome-wide scale.

Regardless of primary lineage-specific causality, we identify a placental expression signature that correlates with developmental heart abnormalities. This connection pertains specifically to marker genes of SynT-I, the syncytiotrophoblast layer facing the maternal blood space, which is highly analogous to the single syncytiotrophoblast layer in the human placenta. This remarkable observation opens up avenues for therapeutic applications, in particular as cell-free placental mRNA is detectable in maternal blood and is predictive of pregnancy complications^51^. Here, we add to this breakthrough by suggesting that placental expression signatures may not only be predictive of syndromic pregnancy complications such as preeclampsia, but may indeed be able to identify specific developmental abnormalities intrinsic to the embryo itself.

In any case, the question remains as to how the placenta affects heart development. Multiple possibilities have been suggested, such as haemodynamics, placenta-produced growth factors, hormones or miRNAs, or oxygen and nutrient supply^9,52^. Many mouse mutations affect the organization of the placental labyrinthine vasculature^53,54^, yet not all of these mutations cause a heart defect. This finding appears to argue against a sole role of haemodynamics, hypoxia, or nutrient deficiencies. The *Atp11a*-mutant phenotype resembles most closely that of *Ly6e*^12^, which encodes a syncytin receptor required for SynT-I fusion. These similarities encompass embryonic lethality in the second half of gestation, ventricular wall thinning, VSD, the majority of placental pathologies, and the fact that both *Atp11a*^−/−^ and *Ly6e*^−/−^ cKO-E mice survive into adulthood. We further build on these insights and identify a SynT-I defect also in TSCs mutant for *Pparg* and *Smg9*, i.e. genes that share a full or even partial placental contribution to the heart defect, respectively. Thereby, we uncover a critical mechanistic link that ties heart development to SynT-I. Together with the establishment of tools that enable a systematic investigation of the impact of the placenta on embryonic development, our study suggests that placentally induced heart defects are a significant contributor to CHDs. These insights provide a paradigm shift that will help transform our understanding of CHDs as the most common birth defect and as a major contributor to pregnancy losses.

## Methods

### Mice

Sperm carrying the *Atp11a*^*tm1a(KOMP)Wtsi*^, *Smg9*^*tm1a(EUCOMM)Wtsi*^ and *Ssr2*^*tm1a(EUCOMM)Wtsi*^ were purchased from the International Mouse Phenotyping Consortium (IMPC) and used for in vitro fertilization of C57BL/6 N (Charles River) oocytes to re-derive the corresponding mouse strains. All strains were maintained as heterozygotes by routine breeding to C57BL/6-Elite (Charles River) mice. For conversion of tm1a into tm1b, tm1c, and tm1d alleles, we used the *Pgk1*-FlpO line (B6.Cg-Tg(Pgk1-flpo)10Sykr/J, strain ID 011065) and the *Sox2*-Cre line (B6.Cg-Edil3 < Tg(Sox2-cre)1Amc > /J, strain ID 008454) as appropriate, which we purchased from Jackson Laboratories. All animal work was conducted with approval by the University of Calgary’s animal care committee, and with appropriate Health Sciences Animal Care Committee (HSACC)-approved animal use protocols in place (protocol number AC18-0191). Mice were housed in IVC cages with 12 hour light-dark cycles under ambient temperature (~22 °C) and humidity conditions. All genotyping primers are provided in Supplementary Table 3.

### Sox2-Flp transgenic line generation

To generate the *Sox2*-Flp line, we obtained the original plasmid used for the generation of the *Sox2*-Cre line from Dr. A. McMahon’s lab, which contains a ~13.7 kb genomic fragment encompassing the *Sox2* promoter that drives expression of the downstream gene in an epiblast-specific manner^25^. We replaced Cre with a FlpO coding sequence using standard recombinant DNA techniques. A *Pme*I-excised *Sox2*-FlpO insert was used for pronuclear injection into fertilized C57BL/6 x (DBA x C57BL/6) oocytes. 5 original transgenic founders were obtained that were propagated as hemizygous lines by mating to C57BL/6-Elite (Charles River) mice. Cell lineage-specific activity of the newly established *Sox2*-Flp line was tested as follows: 1. *Sox2*-FlpO transgenic males were crossed to the RCE:FRT reporter strain (MMRRC Strain #032038-JAX) which harbors the R26R CAG-boosted EGFP (RCE) reporter allele with a FRT-flanked STOP cassette upstream of an enhanced green fluorescent protein (EGFP) gene. After removal of the FRT-flanked STOP cassette by FLP-mediated recombination, EGFP reporter expression is directed to the cells/tissues in which FLP is expressed. 2. In addition, *Sox2*-Flp transgenic males were crossed with heterozygous *Ssr2* tm1a females in which FLP activity is identified by the loss of LacZ expression. 3. Detailed genotyping was performed on fine-dissected tissue to establish full allele conversions. These strategies collectively illustrated FLP activity in all epiblast-derived tissues, resulting in non-mosaic EGFP expression (on mating with the RCE:FRT strain) and complete loss of LacZ expression (on mating to *Ssr2* tm1a/+ females) in all cells of the embryo proper and of the extra-embryonic mesoderm (Fig. 3b, Supplementary Figs. 3, 4). Epiblast-restricted activity of *Sox2*-Flp was given in most instances, with only rare occasions of ectopic expression in trophoblast cells of the junctional zone in one placenta, with <1% of junctional zone cells affected. This was observed with the RCE:FRT reporter but not with the *Ssr2* tm1a reporter alleles. Thus, the *Sox2*-Flp line was established as a novel tool to revert knockout-first alleles into functional alleles in all cells of the embryo proper while leaving the gene knocked out in all, or the vast majority of, trophoblast cells. Two *Sox2*-Flp founder lines (1.27 and 4.2.28) matched these criteria and were used in further study.

### Generation of conceptuses with informative allele combinations

To obtain conceptuses for the initial in-depth analysis of WT, HET, and KO embryos and placentas, we intercrossed heterozygous knockout-first tm1a/+ mice of each strain. To obtain informative conditional knockout conceptuses, we first generated males carrying the necessary allele combinations ([tm1b/+; *Sox2*-Cre/+ or tm1d/+; *Sox2*-Cre/+] and [tm1b/+; *Sox2*-Flp/+]) by intercrossing the corresponding mouse lines. Tm1c/+ and tm1c/tm1c conditional-ready females were generated by crossing tm1a/+ mice with *Pgk1*-FlpO mice. In all subsequent informative matings, the cross was such that transmission of the *Sox2*-Flp and *Sox2*-Cre drivers were from the male to ensure epiblast-specific expression^38^. Crosses were as outlined in Supplementary Fig. 3b; in all crosses, the female is always named first.

The appearance of a vaginal plug was counted as embryonic day (E) 0.5. Pregnant females were routinely dissected on E14.5; embryos were fixed in 4% paraformaldehyde (PFA) overnight at 4 °C. Placentas were bisected, one-half snap-frozen in liquid nitrogen and the other half fixed in 4% PFA alongside the embryo. All genotyping primers are provided in Supplementary Table 3.

### LacZ staining

Embryos and placentas used for LacZ staining were fixed in 0.25% glutaraldehyde and used either for whole-mount staining or for cryo-embedding and sectioning. Embryos and cryosections were stained for beta-galactosidase activity using standard protocols^55^.

### µCT analysis

Embryos were fixed overnight in 4% PFA and stored in PBS. Fixed embryos were then stained overnight in Lugol’s iodine (2.5% w/v I_2_Kl) dye and embedded in 1% agarose immediately before scanning. Images were obtained on a ZEISS Xradia Versa 520 X-ray microscope 186 (Carl Zeiss AG, Oberkochen, Germany) with the following settings: ×0.4 objective, 50 kV, 4 W, Binning 2, exposure: 2 s. The resolution obtained was 10-12.89 µm/pixel. Reconstruction was completed by the ZEISS XMReconstructor software and converted to TIFF files with ZEISS XMcontroller software.

Segmentation of TIFF images were completed with Slicer (version 4.11.20210226) with the MarkupsTOModel and SegmentEditorExtraEffects plugins. Measurements taken included: ventricular space, ventricular muscle, liver volume, septal hole size, ventricular thickness, compact layer thickness, and trabecular layer thickness (see Supplementary Note). All measurements were normalized to embryo length. Statistical differences were calculated in GraphPad Prism software (9.4.1). All adjusted *p* values (*padj*) are provided in Supplementary Data 1.

### Histology

Placentas were bisected along the midline and one half fixed in 4% paraformaldehyde for histology, the other half snap-frozen for RNA extraction. Fixed tissues were processed for routine paraffin histology, and serial 7 µm sections produced using a Leica rotary microtome. Sections through the midline of the placenta were identified by the insertion of the umbilical cord. For assessment of placental layers and fetal/maternal blood space areas, at least three sections, positioned 70 µm apart (i.e., 10 consecutive sections apart), were analyzed per placenta. In situ hybridizations using a probe against *Tpbpa* were performed as previously described^8,56^. For immunostaining, sections were deparaffinised in xylene and processed through an ethanol series to PBS. Antigen retrieval was performed by boiling in 10 mM Na-citrate pH 6.0 buffer in a pressure cooker followed by blocking in PBS, 0.5% BSA, 0.1% Tween-20. Antibodies used were against MCT1 (1:200, Sigma AB1286-I), MCT4 (1:200, Sigma AB3314P), RXRa (1:100, Abcam ab125001) and phospho-histone H3S10 (1:200, Millipore-Sigma #06-570). Primary antibodies were detected with appropriate AlexaFluor488- or AlexaFluor568-conjugated secondary antibodies (1:500, ThermoFisher). Counterstaining was performed with 4′,6-diamidino-2-phenylindole (Sigma D9542), slides were mounted with Fluoromount-G (SouthernBiotech) mounting medium and imaged at a Leica DMRE epifluorescence microscope. In the case of the immunofluorescence stainings for beta-galactosidase (LacZ) and MCT1/4, tissue was fixed in 4% paraformaldehyde, embedded in OCT, and cryosectioned. To avoid cross-reactivity with the MCT1 and MCT4 antibodies, two different antibodies against beta-galactosidase were used: Cell Signaling #27198 (1:200) and Abcam ab9361 (1:200). For LacZ/MCT4 double staining, MCT4 was detected with a horseradish peroxidase-conjugated anti-rabbit secondary antibody (1:100, ThermoFisher 31460) followed by color reaction with DAB substrate (ThermoFisher 34002) and counterstaining with nuclear fast red (Sigma N3020). Image analyses of area and perimeter measurements were performed in ImageJ (1.53). Statistical differences were calculated in GraphPad Prism software (9.4.1). All *p*-values are provided in Supplementary Data 1.

### RNA extraction and RT-qPCRs

Total RNA was extracted from snap-frozen tissues or cultured cells using either the *mir*Vana^TM^ micro-RNA isolation kit (ThermoFisher AM1560) according to the manufacturer’s instructions, or using TRI reagent (Sigma T9424). 1-2 µg of total RNA was used for cDNA synthesis with RevertAid H-Minus reverse transcriptase (Thermo Scientific EP0451) and a mix of oligo-d(T)_18_ (ThermoFisher FERSO132) and random primers (ThermoFisher FERSO142). Quantitative (q)PCR was performed using the QuantiFast (Qiagen 25057), QuantiNova (Qiagen 208057) or SsoAdvanced Universal SYBR Green Supermix (BioRad 1725274) and intron-spanning primer pairs on a QuantStudio 3 (ThermoFisher) or CFX384 (Bio-Rad) real-time PCR thermocycler. All primers were tested for efficiency and correct product amplification prior to use. In the few cases where primers were non-intron-spanning, RNA was DNAse treated before cDNA synthesis. Expression levels were normalized against housekeeping gene *Sdha* and are displayed as mean relative to the WT control samples; error bars indicate standard error of the means (SEM) of at least three independent biological replicates. Where appropriate, Student’s *t* test or ANOVA was performed to calculate the statistical significance of expression differences (*p* < 0.05) using GraphPad Prism software (9.4.1); all *p*-values are provided in Supplementary Data 1.

### RNA-sequencing

RNA for transcriptomic analysis was isolated using the *mir*Vana^TM^ micro-RNA isolation kit (ThermoFisher AM1560). Library construction with NEB Ultra II Directional RNA Library Prep and sequencing on an Illumina NovaSeq 6000 (50 bp paired-end) was completed by the University of Calgary’s Center for Health Genomics and Informatics facility. Reads were aligned with STAR^57^ (2.6.1a_08-27) to the reference mouse genome (GRCm38/mm10 from Ensembl FTP https://uswest.ensembl.org/info/data/ftp/index.html). Count tables were assembled with htseq-count (bioconda 2018.11) using the reverse strand and non-unanimous settings, along with all other default settings^58^, resulting in 27M-46M gene counts per sample. Differential gene expression, sample visualization, and estimation of trophoblast cell proportions were completed in R (version 3.4.3 and 4.1.0) (https://www.R-project.org/). Prior to collapsing lanes, intra-lane variability was assessed with centered-log ratio transformed scaled biplots. No lane replicates contained high enough variance to be located in different PCA quadrants, all lanes were then aggregated. Count tables for each mouse line (*Atp11a*, *Smg9,* and *Ssr2*) were adjusted for litter differences with the limma package^59^. Functions from DESeq2 (1.32.0), RColorBrewer (1.1-2), gplots (3.1.1), ggplot2 (3.3.5), and biomaRt (2.48.3) were used to generate regularized log-transformed PCA’s, and heatmaps of log_2_-centered differentially expressed genes^60^. Differential expression analysis was performed with DESeq2 (1.32.0) and EdgeR (3.34.1), and genes were called differentially expressed (DE) between wild-type and KO placentas if they were detected by both packages (FDR < 0.1, FC >|1.5|)^60,61^. Venn Diagrams showing DE gene overlaps were created with the package VennDiagram^62^ (1.7.0). Lastly, to estimate cell proportions, the E14.5 placenta single nuclei RNAseq data published by Marsh and Blelloch^39^ was used. Devtools (2.4.2), loomR (0.2.1.9000), and stringr (1.4.0) were packages required to access and segment this data (https://github.com/r-lib/devtools; https://github.com/tidyverse/stringr). The single nuclei RNAseq data was integrated with our own E14.5 whole tissue placenta RNA sequencing and cell populations were estimated with BisqueRNA^40^ (1.0.5). Proportions were plotted in Excel and significant differences were detected with unpaired student’s *t* tests in GraphPad Prism (version 9.4.1).

### Trophoblast stem cell culture and KO generation

The wild-type blastocyst-derived TS-Rs26 TSC line (a kind gift of the Rossant lab, Toronto, Canada) was cultured as described previously^63,64^. Briefly, cells were grown in 20% fetal bovine serum (FBS) (Wisent Bioproducts, 098-150), 1 mM sodium pyruvate (Invitrogen, 11360–039), 1× anti-mycotic/antibiotic (Invitrogen, 15240062), 50 μM 2-mercaptoethanol (Invitrogen, 31350010), 37.5 ng/ml bFGF (Cambridge Stem Cell Institute), and 1 μg/ml heparin (Sigma) in RPMI 1640 with l-glutamine (ThermoFisher Scientific, 11875093), with 70% of the medium pre-conditioned on mouse embryonic fibroblasts. Differentiation was induced by culturing in media lacking bFGF, Heparin, and embryonic fibroblast-conditioned medium.

For generation of CRISPR/Cas9-mediated knockout TSCs, gRNAs that result in exon deletion and frameshift mutations in the remaining transcript sequence were designed using the http://crispor.tefor.net/ interface, chosen on the basis of high-specificity scores. All gRNA sequences are provided in Supplementary Table 3. gRNA sequences were cloned into the Cas9.2 A.EGFP plasmid (Plasmid #48138 Addgene) and sequence-verified. Transfection was carried out with Lipofectamine 2000 (ThermoFisher Scientific 11668019) reagent according to the manufacturer’s protocol. EGFP-positive cells were single cell-sorted by the Flow Cytometry Core Facility at the University of Calgary into 96-well plates using BD FACSDiva Software (8.0.1 build 2014 07 03 11 47) and Firmware (version 1.3) (Supplementary Fig. 11) and expanded for KO TSC clone generation. Deletion of the targeted exon was confirmed by genotyping PCRs using primers spanning the deleted exon (expected product shorter than WT sequence), and positioned within the deleted exon (negative control). EGFP-negative single cell-sorted cells from the same transfected TSC pools were used to generate WT control clones. 3-11 independent single cell-derived KO clones and 4-8 single cell-derived WT clones isolated alongside in every targeting round were analyzed for each gene mutation.

### Cell fusion analysis

To assess fusogenic capacity of *Atp11a*^−/−^ compared to WT TSCs, TSCs were plated on coverslips and differentiated for 4 days in media lacking bFGF, Heparin and embryonic fibroblast-conditioned medium. Cells were rinsed in PBS, fixed in 4% PFA for 10 minutes and permeabilised with PBS, 0.1% Triton X-100 for 30 minutes. Membrane-specific staining was performed with an antibody against ZO-1 (1:100, ThermoFisher 339100) followed by detection with an appropriate anti-mouse AlexaFluor568-coupled secondary antibody (1:500, ThermoFisher) and DAPI counterstaining. Nuclei surrounded by continuous or largely continuous ZO-1 staining were counted as single cells, those that lacked a discernible surrounding by ZO-1 stained cell membrane were counted as syncytial. Counts were performed in a blinded fashion of two independent WT and two independent KO clones, analyzing a total of 2052 and 1640 nuclei, respectively. Genotype-dependent differences were determined by student’s *t* test.

### TSC proliferation and differentiation analysis

Three KO and 4–5 wild-type TSC clones of each mutant TSC line (*Atp11a*, *Smg9,* and *Pparg*) were passaged twice in full TSC media before plating was set up for analysis: For the shorter time points (2–4 days), 50,000 cells were seeded following quantification with an automated cell counter (Luna II, Logosbio) into a six-well plate in routine stem cell maintenance (STEM) or in differentiation conditions, and cells were counted on days 2 and 4. A different set of plates was also seeded in the same conditions for RNA isolations. For extended differentiation assays, 25,000 cells were seeded for the 6-day time point and 15,000 cells for the 8D time point. Two-way ANOVA or unpaired *t* tests in GraphPad Prism (9.4.1) were used to compare proliferation rates over time, the area under each growth curve, and the expression of trophoblast-derived cell type markers.

### Reporting summary

Further information on research design is available in the Nature Portfolio Reporting Summary linked to this article.

## Supplementary information

Supplementary Information

Description of Additional Supplementary Files

Supplementary Data 1

Supplementary Movie 1

Supplementary Movie 2

Supplementary Movie 3

Reporting Summary

## Data Availability

The sequencing data generated in this study have been deposited in the GEO database under accession number GSE204859. Source data are provided with this paper.

## Source data

Source Data

## References

[1] Hoffman J (2013). The global burden of congenital heart disease. Cardiovasc. J. Afr..

[2] Jorgensen M, McPherson E, Zaleski C, Shivaram P, Cold C (2014). Stillbirth: the heart of the matter. Am. J. Med. Genet. A.

[3] Richards AA, Garg V (2010). Genetics of congenital heart disease. Curr. Cardiol. Rev..

[4] Yasuhara J, Garg V (2021). Genetics of congenital heart disease: a narrative review of recent advances and clinical implications. Transl. Pediatr..

[5] Morton SU, Quiat D, Seidman JG, Seidman CE (2022). Genomic frontiers in congenital heart disease. Nat. Rev. Cardiol..

[6] Pierpont ME (2018). Genetic basis for congenital heart disease: revisited: a scientific statement from the american heart association. Circulation.

[7] McCulley DJ, Black BL (2012). Transcription factor pathways and congenital heart disease. Curr. Top. Dev. Biol..

[8] Perez-Garcia V (2018). Placentation defects are highly prevalent in embryonic lethal mouse mutants. Nature.

[9] Camm EJ, Botting KJ, Sferruzzi-Perri AN (2018). Near to One’s heart: the intimate relationship between the placenta and fetal heart. Front. Physiol..

[10] Courtney JA, Cnota JF, Jones HN (2018). The role of abnormal placentation in congenital heart disease; cause, correlate, or consequence?. Front. Physiol..

[11] Barak Y (1999). PPAR gamma is required for placental, cardiac, and adipose tissue development. Mol. Cell.

[12] Langford MB, Outhwaite JE, Hughes M, Natale DRC, Simmons DG (2018). Deletion of the Syncytin A receptor Ly6e impairs syncytiotrophoblast fusion and placental morphogenesis causing embryonic lethality in mice. Sci. Rep..

[13] Adams RH (2000). Essential role of p38alpha MAP kinase in placental but not embryonic cardiovascular development. Mol. Cell.

[14] Raffel GD (2009). Ott1 (Rbm15) is essential for placental vascular branching morphogenesis and embryonic development of the heart and spleen. Mol. Cell Biol..

[15] Maruyama EO (2016). Extraembryonic but not embryonic SUMO-specific protease 2 is required for heart development. Sci. Rep..

[16] Hatano N (2003). Essential role for ERK2 mitogen-activated protein kinase in placental development. Genes Cells.

[17] Torregrosa-Carrion R (2021). Adhesion G protein-coupled receptor Gpr126/Adgrg6 is essential for placental development. Sci. Adv..

[18] Wang J, Mager J, Schnedier E, Magnuson T (2002). The mouse PcG gene eed is required for Hox gene repression and extraembryonic development. Mamm. Genome.

[19] Christie GR (2005). The dual-specificity protein phosphatase DUSP9/MKP-4 is essential for placental function but is not required for normal embryonic development. Mol. Cell Biol..

[20] Ayadi A (2012). Mouse large-scale phenotyping initiatives: overview of the European Mouse Disease Clinic (EUMODIC) and of the wellcome trust sanger institute mouse genetics Project. Mamm. Genome.

[21] de Angelis MH (2015). Analysis of mammalian gene function through broad-based phenotypic screens across a consortium of mouse clinics. Nat. Genet..

[22] White JK (2013). Genome-wide generation and systematic phenotyping of knockout mice reveals new roles for many genes. Cell.

[23] Adams D (2013). Bloomsbury report on mouse embryo phenotyping: recommendations from the IMPC workshop on embryonic lethal screening. Dis. Models Mech..

[24] Dickinson ME (2016). High-throughput discovery of novel developmental phenotypes. Nature.

[25] Hayashi S, Lewis P, Pevny L, McMahon AP (2002). Efficient gene modulation in mouse epiblast using a Sox2Cre transgenic mouse strain. Mech. Dev..

[26] Skarnes WC (2011). A conditional knockout resource for the genome-wide study of mouse gene function. Nature.

[27] Mohun T (2013). Deciphering the mechanisms of developmental disorders (DMDD): a new programme for phenotyping embryonic lethal mice. Dis. Model. Mech..

[28] Wang J (2018). Proteomic analysis and functional characterization of P4-ATPase phospholipid flippases from murine tissues. Sci. Rep..

[29] Bono F (2014). Juggling key players in NMD initiation. Structure.

[30] Hartmann E (1993). A tetrameric complex of membrane proteins in the endoplasmic reticulum. Eur. J. Biochem..

[31] Pfeffer S (2017). Dissecting the molecular organization of the translocon-associated protein complex. Nat. Commun..

[32] Shaheen R (2016). Mutations in SMG9, encoding an essential component of nonsense-mediated decay machinery, cause a multiple congenital anomaly syndrome in humans and mice. Am. J. Hum. Genet..

[33] Wilson R (2016). Highly variable penetrance of abnormal phenotypes in embryonic lethal knockout mice. Wellcome Open Res..

[34] Makita T (2001). A developmental transition in definitive erythropoiesis: erythropoietin expression is sequentially regulated by retinoic acid receptors and HNF4. Genes Dev..

[35] Ueno M (2013). c-Met-dependent multipotent labyrinth trophoblast progenitors establish placental exchange interface. Dev. Cell.

[36] Savolainen SM, Foley JF, Elmore SA (2009). Histology atlas of the developing mouse heart with emphasis on E11.5 to E18.5. Toxicol. Pathol..

[37] Wang J, Vasaikar S, Shi Z, Greer M, Zhang B (2017). WebGestalt 2017: a more comprehensive, powerful, flexible and interactive gene set enrichment analysis toolkit. Nucleic Acids Res..

[38] Hayashi S, Tenzen T, McMahon AP (2003). Maternal inheritance of Cre activity in a Sox2Cre deleter strain. Genesis.

[39] Marsh B, Blelloch R (2020). Single nuclei RNA-seq of mouse placental labyrinth development. Elife.

[40] Jew B (2020). Accurate estimation of cell composition in bulk expression through robust integration of single-cell information. Nat. Commun..

[41] Caldas H (2005). Placental defects are associated with male lethality in bare patches and striated embryos deficient in the NAD(P)H steroid dehydrogenase-like (NSDHL) enzyme. Mol. Genet Metab..

[42] Jiang F, Herman GE (2006). Analysis of Nsdhl-deficient embryos reveals a role for Hedgehog signaling in early placental development. Hum. Mol. Genet..

[43] Nadeau V, Charron J (2014). Essential role of the ERK/MAPK pathway in blood-placental barrier formation. Development.

[44] Ochiai Y, Suzuki C, Segawa K, Uchiyama Y, Nagata S (2022). Inefficient development of syncytiotrophoblasts in the Atp11a-deficient mouse placenta. Proc. Natl Acad. Sci. USA.

[45] Jones HN (2015). Hypoplastic left heart syndrome is associated with structural and vascular placental abnormalities and leptin dysregulation. Placenta.

[46] Rychik J (2018). Characterization of the placenta in the newborn with congenital heart disease: distinctions based on type of cardiac malformation. Pediatr. Cardiol..

[47] Matthiesen NB (2016). Congenital heart defects and indices of placental and fetal growth in a nationwide study of 924 422 liveborn infants. Circulation.

[48] Kelly RG (2012). The second heart field. Curr. Top. Dev. Biol..

[49] Nagy A (1990). Embryonic stem cells alone are able to support fetal development in the mouse. Development.

[50] Tallquist MD, Soriano P (2000). Epiblast-restricted Cre expression in MORE mice: a tool to distinguish embryonic vs. extra-embryonic gene function. Genesis.

[51] Rasmussen M (2022). RNA profiles reveal signatures of future health and disease in pregnancy. Nature.

[52] Maslen CL (2018). Recent advances in placenta-heart interactions. Front. Physiol..

[53] Watson ED, Cross JC (2005). Development of structures and transport functions in the mouse placenta. Physiology (Bethesda).

[54] Woods L, Perez-Garcia V, Hemberger M (2018). Regulation of placental development and its impact on fetal growth-new insights from mouse models. Front. Endocrinol. (Lausanne).

[55] Eid R, Koseki H, Schughart K (1993). Analysis of LacZ reporter genes in transgenic embryos suggests the presence of several cis-acting regulatory elements in the murine Hoxb-6 gene. Dev. Dyn..

[56] Hemberger M, Nozaki T, Masutani M, Cross JC (2003). Differential expression of angiogenic and vasodilatory factors by invasive trophoblast giant cells depending on depth of invasion. Dev. Dyn..

[57] Dobin A (2013). STAR: ultrafast universal RNA-seq aligner. Bioinformatics.

[58] Anders S, Pyl PT, Huber W (2015). HTSeq–a Python framework to work with high-throughput sequencing data. Bioinformatics.

[59] Ritchie ME (2015). limma powers differential expression analyses for RNA-sequencing and microarray studies. Nucleic Acids Res..

[60] Love MI, Huber W, Anders S (2014). Moderated estimation of fold change and dispersion for RNA-seq data with DESeq2. Genome Biol..

[61] Robinson MD, McCarthy DJ, Smyth GK (2010). edgeR: a Bioconductor package for differential expression analysis of digital gene expression data. Bioinformatics.

[62] Chen H, Boutros PC (2011). VennDiagram: a package for the generation of highly-customizable Venn and Euler diagrams in R. BMC Bioinform..

[63] Tanaka S, Kunath T, Hadjantonakis AK, Nagy A, Rossant J (1998). Promotion of trophoblast stem cell proliferation by FGF4. Science.

[64] Murray A, Sienerth AR, Hemberger M (2016). Plet1 is an epigenetically regulated cell surface protein that provides essential cues to direct trophoblast stem cell differentiation. Sci. Rep..

