# Supplementary material for: Defects in placental syncytiotrophoblast cells are a common cause of developmental heart disease
Source: Nat Commun. 2023 Mar 1;14:1174. doi: 10.1038/s41467-023-36740-5 (PMC9978031; doi:10.1038/s41467-023-36740-5)
Supplement: Supplementary file 1 — Supplementary Information [file 41467_2023_36740_MOESM1_ESM.pdf]

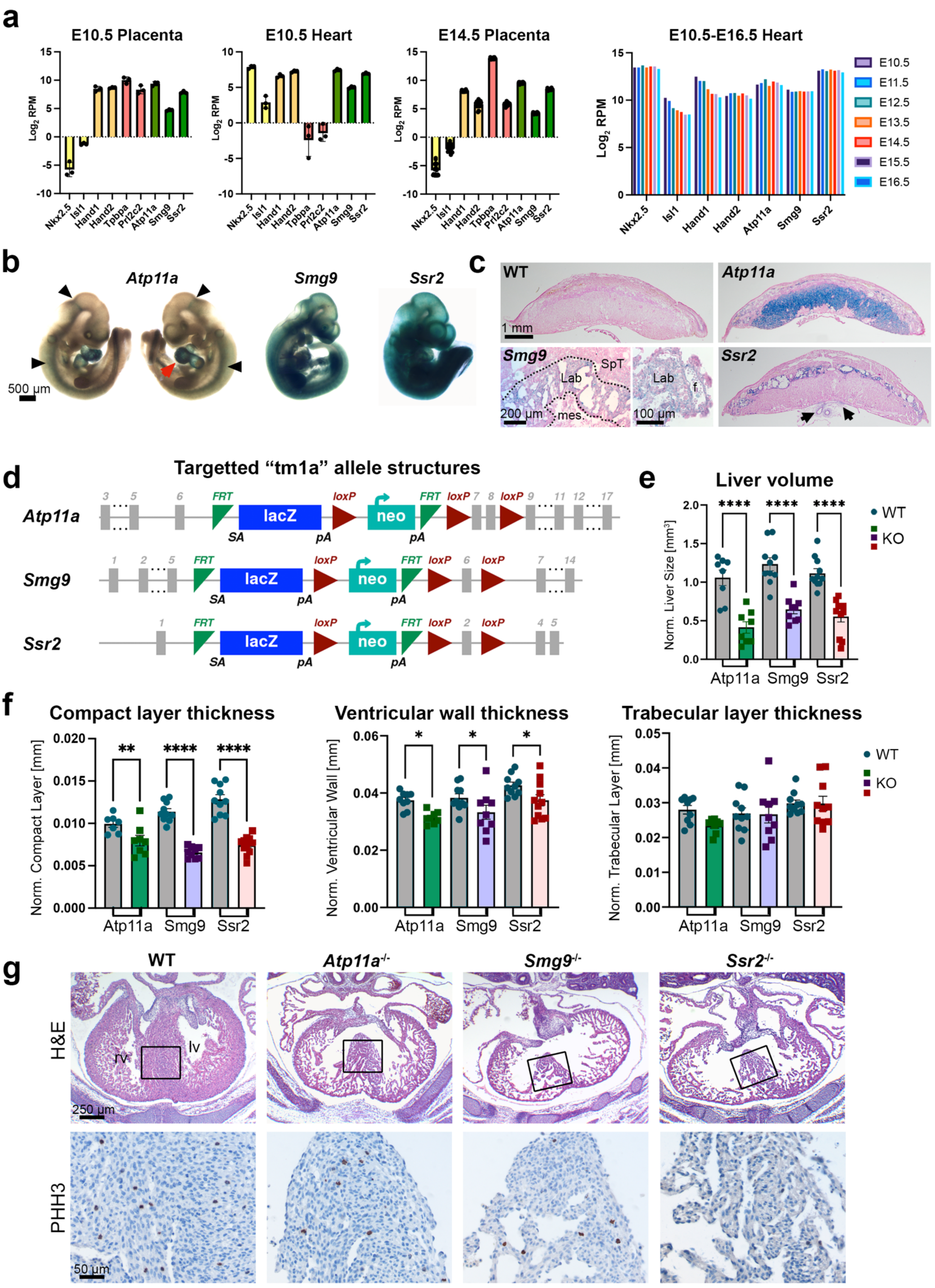
a
E10.5 Placenta
E10.5 Heart
E14.5 Placenta
E10.5-E16.5 Heart
Log2 RPM
Nkx2.5
Isl1
Hand1
Hand2
Tpbpa
Prl2c2
Atp11a
Smg9
Ssr2
E10.5
E11.5
E12.5
E13.5
E14.5
E15.5
E16.5
b
Atp11a
Smg9
Ssr2
500 µm
c
WT
Atp11a
Smg9
Ssr2
1 mm
SpT
Lab
mes
200 µm
100 µm
f
d
Targetted "tm1a" allele structures
FRT
lacZ
loxP
neo
SA
pA
e
Liver volume
Norm. Liver Size [mm³]
WT
KO
f
Compact layer thickness
Norm. Compact Layer [mm]
Ventricular wall thickness
Norm. Ventricular Wall [mm]
Trabecular layer thickness
Norm. Trabecular Layer [mm]
g
WT
Atp11a-/-
Smg9-/-
Ssr2-/-
H&E
PHH3
rv
lv
250 µm
50 µm

Supplementary Figure 1. Expression and phenotypic analysis of selected mouse mutant genes.

a RNA-seq data of E10.5 hearts and placentas, E14.5 placentas and across an E10.5-E16.5 developmental time course of heart development[1] show that *Atp11a*, *Smg9* and *Ssr2* are expressed in both organs. Genes colour-coded in yellow, orange and red are heart and placenta markers that are displayed for comparative reference. Data are displayed as mean +/- SEM from: E10.5 n=3 and E14.5 n=23 samples. **b** Whole-mount LacZ staining of E10.5 embryos heterozygous for the tm1a alleles of *Atp11a*, *Smg9* and *Ssr2*, revealing their endogenous expression pattern. Black arrowheads point to distinct *Atp11a* expression at the midbrain-hindbrain boundary and in the notochord, the red arrowhead points to high expression in the heart ventricles. **c** LacZ staining of E14.5 (WT, $Atp11a^{+/-}$, $Ssr2^{+/-}$) and E10.5 ($Smg9^{+/-}$) placental cryosections. *Smg9* expression decreases during gestation and was not detectable at E14.5 by LacZ staining of cryosections. Two different magnification views are shown for E10.5, the developing labyrinth (Lab) is outlined by dotted lines. Syncytiotrophoblast cells are positive for LacZ, whereas extraembryonic mesoderm (mes.) is negative. For *Ssr2*, black arrows point to expression in extra-embryonic mesoderm of the umbilical cord and fetal endothelial cells. SpT = spongiotrophoblast; f = fetal vessel. Images are representative of at least 3 placentas per genotype. **d** Allele structure and targeted exons, flanked by LoxP sites, of tm1a knockout-first alleles for *Atp11a*, *Smg9* and *Ssr2*. **e** Liver size measurements reveals a significant size reduction in KO embryos. Statistics: One-way ANOVA with Holm-Šídák's multiple comparisons test. *** $p<0.001$, **** $p<0.0001$. **f** Additional measurements of heart structures by segmentation of 3D µCT imaging data. Significance was evaluated using one-way ANOVA with Holm-Šídák's multiple comparisons test. * $p<0.05$, ** $p<0.01$, **** $p<0.0001$. Data in **e-f** are displayed as mean +/- SEM from *Atp11a*: WT n=7, KO n=8, *Smg9*: WT n=10, KO n=9; *Ssr2*: WT= 10, KO n=11 samples. **g** H&E stainings and immunohistochemical detection of phospho-histone H3 at serine 10 (PHH3) on paraffin sections through the heart of WT and KO E14.5 embryos, revealing severe VSDs and myocardial wall thinning in all mutants, possibly due to lower cardiomyocyte proliferation rates. The rectangle identifies the region shown in the PHH3 stainings. Lv=left ventricle, rv=right ventricle. Images are representative of 3 samples per genotype. Source data are provided as a Source Data file. All exact *p*-values are provided in Supplementary Data 1.

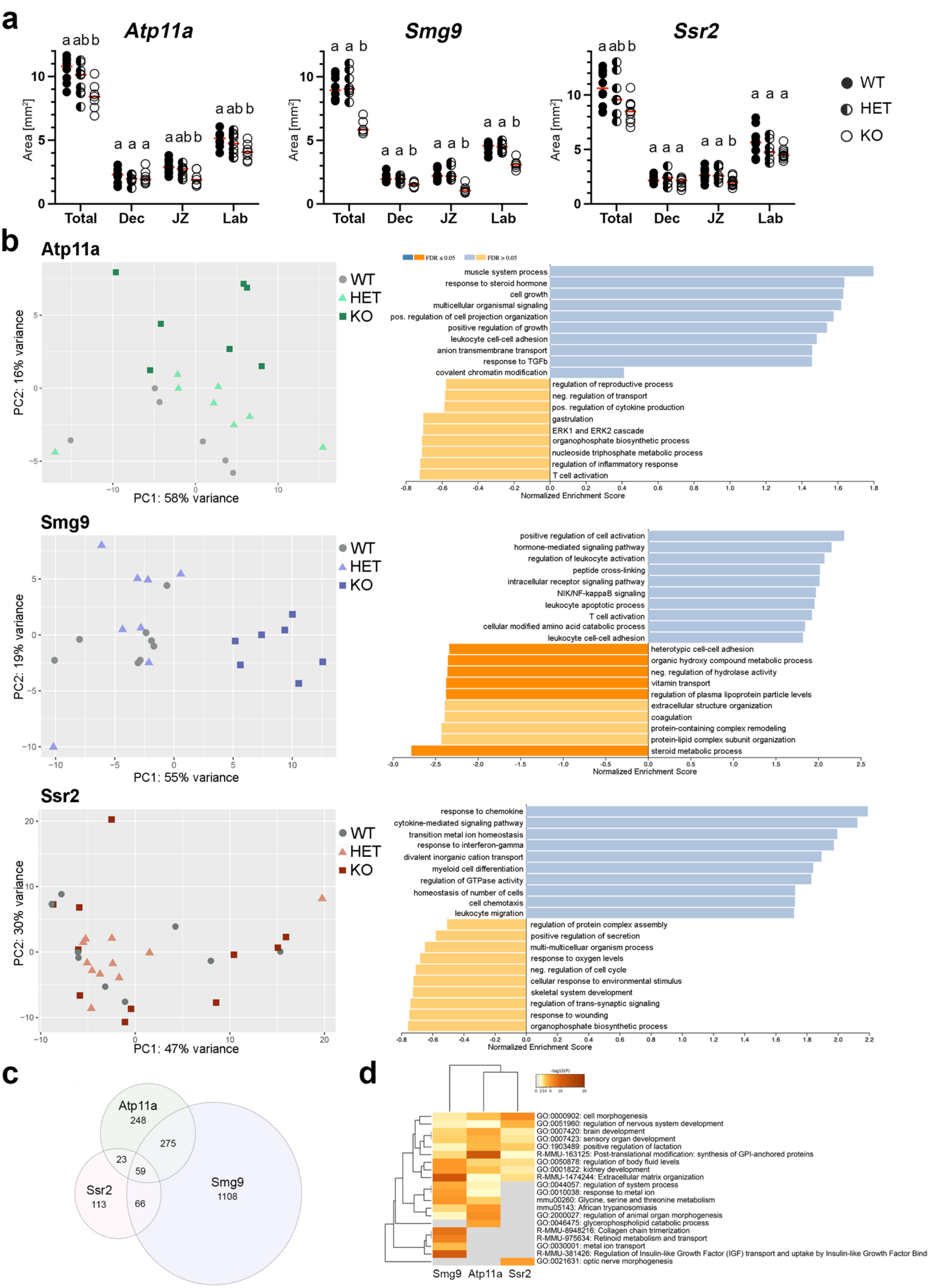
a
Atp11a
Smg9
Ssr2
Area [mm²]
Total
Dec
JZ
Lab
WT
HET
KO
b
Atp11a
PC1: 58% variance
PC2: 16% variance
FDR ≤ 0.05
FDR > 0.05
muscle system process
response to steroid hormone
cell growth
multicellular organismal signaling
pos. regulation of cell projection organization
positive regulation of growth
leukocyte cell-cell adhesion
anion transmembrane transport
response to TGFb
covalent chromatin modification
regulation of reproductive process
neg. regulation of transport
pos. regulation of cytokine production
gastrulation
ERK1 and ERK2 cascade
organophosphate biosynthetic process
nucleoside triphosphate metabolic process
regulation of inflammatory response
T cell activation
Normalized Enrichment Score
Smg9
PC1: 55% variance
PC2: 19% variance
positive regulation of cell activation
hormone-mediated signaling pathway
regulation of leukocyte activation
peptide cross-linking
intracellular receptor signaling pathway
NIK/NF-kappaB signaling
leukocyte apoptotic process
T cell activation
cellular modified amino acid catabolic process
leukocyte cell-cell adhesion
heterotypic cell-cell adhesion
organic hydroxy compound metabolic process
neg. regulation of hydrolase activity
vitamin transport
regulation of plasma lipoprotein particle levels
extracellular structure organization
coagulation
protein-containing complex remodeling
protein-lipid complex subunit organization
steroid metabolic process
Normalized Enrichment Score
Ssr2
PC1: 47% variance
PC2: 30% variance
response to chemokine
cytokine-mediated signaling pathway
transition metal ion homeostasis
response to interferon-gamma
divalent inorganic cation transport
myeloid cell differentiation
regulation of GTPase activity
homeostasis of number of cells
cell chemotaxis
leukocyte migration
regulation of protein complex assembly
positive regulation of secretion
multi-multicellular organism process
response to oxygen levels
neg. regulation of cell cycle
cellular response to environmental stimulus
skeletal system development
regulation of trans-synaptic signaling
response to wounding
organophosphate biosynthetic process
Normalized Enrichment Score
c
Atp11a
248
275
23
59
Ssr2
113
66
Smg9
1108
d
-log10(P)
GO:0000902: cell morphogenesis
GO:0051960: regulation of nervous system development
GO:0007420: brain development
GO:0007423: sensory organ development
GO:1903489: positive regulation of lactation
R-MMU-163125: Post-translational modification: synthesis of GPI-anchored proteins
GO:0050878: regulation of body fluid levels
GO:0001822: kidney development
R-MMU-1474244: Extracellular matrix organization
GO:0044057: regulation of system process
GO:0010038: response to metal ion
mmu00260: Glycine, serine and threonine metabolism
mmu05143: African trypanosomiasis
GO:2000027: regulation of animal organ morphogenesis
GO:0046475: glycerophospholipid catabolic process
R-MMU-8948216: Collagen chain trimerization
R-MMU-975634: Retinoid metabolism and transport
GO:0030001: metal ion transport
R-MMU-381426: Regulation of Insulin-like Growth Factor (IGF) transport and uptake by Insulin-like Growth Factor Bind
GO:0021631: optic nerve morphogenesis
Smg9
Atp11a
Ssr2

Supplementary Figure 2. Placental phenotype and transcriptome-wide gene expression analysis.

a Area measurements of wild-type (WT), heterozygous (HET) and knockout (KO) placentas for each of the three mouse mutant lines. Measurements were performed on three sections around the placental midline that were stained for *Tpbpa* expression by *in situ* hybridization. *Tpbpa* demarcates the junctional zone (JZ) and thereby allows for an unambiguous determination of the dimensions of the decidua (Dec) and labyrinth (Lab). Statistical significance was determined using two-way ANOVA with Tukey's multiple comparisons test on *Atp11a*: WT n=8, HET = 9, KO n=7, *Smg9*: WT n=8, HET n = 8, KO n=6; *Ssr2*: WT= 10, HET = 7, KO n=10 samples. Letters depict statistical similarities (same letter) and differences (discrepant letter). All exact *p*-values are provided in Supplementary Data 1. **b** Principal component analysis (PCA) of placental transcriptomes for each genotype (WT, HET, KO) in the three mutant lines. RNA-seq data included that passed quality control analyses were of 6-11 placentas of each genotype for the three mutant lines (75 RNA-seq datasets in total). Differential gene expression was determined using DESeq2 and EdgeR. Enriched gene ontology terms were determined with the WEB-based GEne SeT AnaLysis (Webgestalt) toolkit using significantly differentially expressed genes (FDR >0.1) with a fold change ≥ |1.5|. **c** Overlap of differentially expressed genes between *Atp11a*, *Smg9* and *Ssr2* KO placentas. **d** Gene ontology enrichments of differentially expressed genes shared between KO placentas of the three mouse lines, determined using Metascape (https://metascape.org). Source data are provided as a Source Data file.

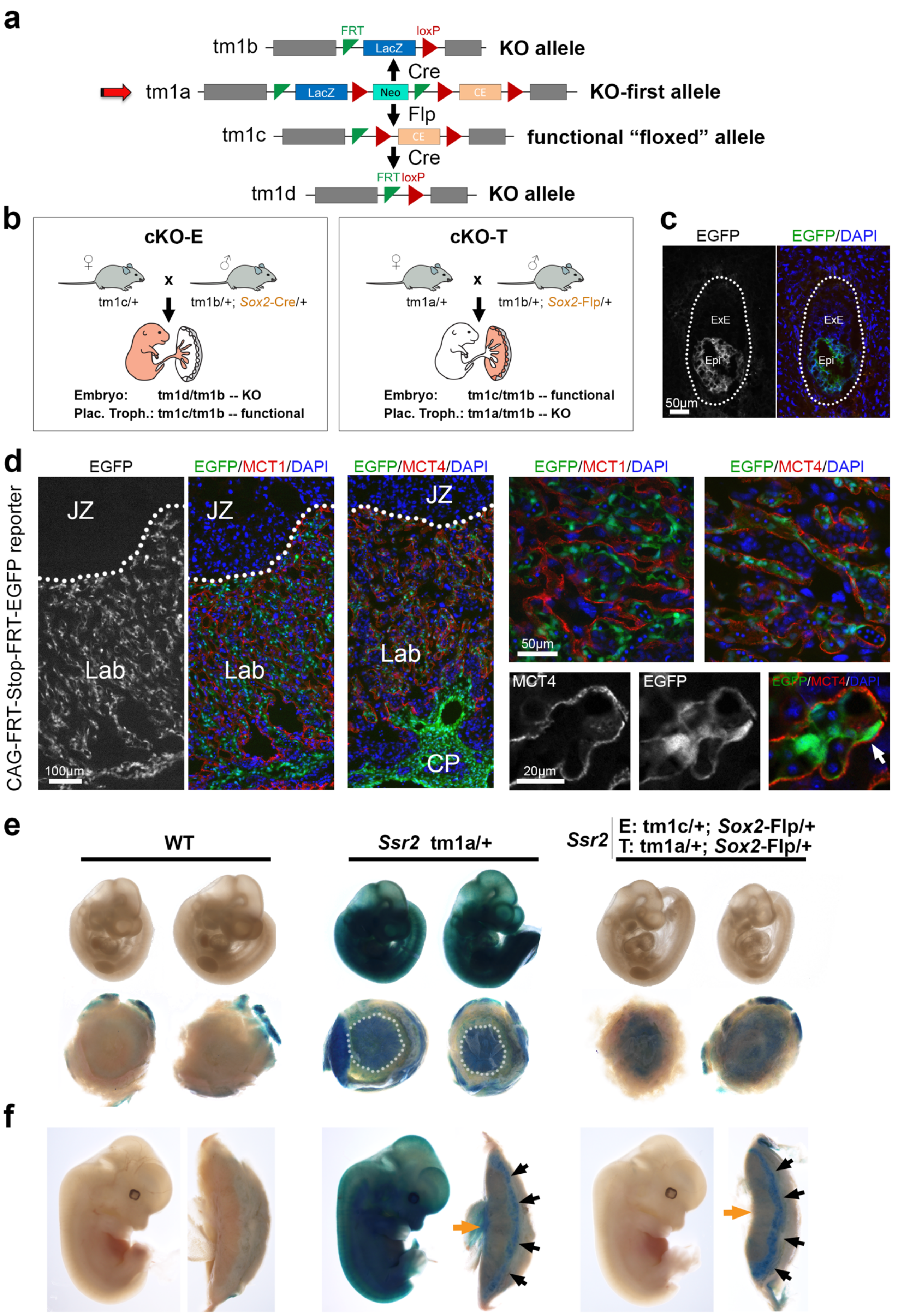
a
tm1b
FRT
LacZ
loxP
KO allele
Cre
tm1a
LacZ
Neo
CE
KO-first allele
Flp
tm1c
CE
functional "floxed" allele
Cre
tm1d
FRT loxP
KO allele
b
cKO-E
tm1c/+
x
tm1b/+; Sox2-Cre/+
Embryo: tm1d/tm1b -- KO
Plac. Troph.: tm1c/tm1b -- functional
cKO-T
tm1a/+
x
tm1b/+; Sox2-Flp/+
Embryo: tm1c/tm1b -- functional
Plac. Troph.: tm1a/tm1b -- KO
c
EGFP
EGFP/DAPI
ExE
Epi
50µm
d
CAG-FRT-Stop-FRT-EGFP reporter
EGFP
EGFP/MCT1/DAPI
EGFP/MCT4/DAPI
JZ
Lab
CP
100µm
50µm
MCT4
EGFP
20µm
e
WT
Ssr2 tm1a/+
Ssr2
E: tm1c/+; Sox2-Flp/+
T: tm1a/+; Sox2-Flp/+
f

Supplementary Figure 3. Conditional knockout strategies.

a Diagram of the EUCOMM/KOMP allele structures. Starting from the knockout-first tm1a allele (red arrow) in which LacZ serves as a readout of endogenous gene expression, exposure to Cre recombinase generates a constitute KO tm1b allele. By contrast, exposure to FLP recombinase excises the LacZ-Neo cassette and generates a functional, “conditional-ready” tm1c allele, thereby restoring gene function in tissues where Flp is expressed. Subjecting the tm1c allele to Cre recombinase generates a constitutive KO tm1d allele. CE = critical exon. **b** Breeding strategy to generate embryo-specific (cKO-E) and trophoblast-specific (cKO-T) conditional knockout conceptuses. The pink shading demarcates gene ablation either in the embryo and in the allantoic (extra-embryonic) mesoderm-derived endothelial cells of fetal blood vessels in the placental labyrinth for cKO-E, or in the trophoblast components of the placenta only (cKO-T). The rendering of the embryo-placenta schematic is from Perez-Garcia et al., Placentation defects are highly prevalent in embryonic lethal mouse mutants. Nature 555, 463-468 (2018). **c** Cryosection of an E5.5 conceptus derived from a cross between a FLP reporter female and *Sox2*-Flp male. Epiblast (Epi) cells express enhanced green fluorescent protein (EGFP) as an indicator of FLP activity, whereas trophoblast cells of the extra-embryonic ectoderm (ExE) remain EGFP-negative. **d** E12.5 placentas of FLP reporter females that express EGFP in cells in which FLP was active, as a readout of *Sox2*-Flp transgene expression. Overview images show EGFP-positive cells in the placental labyrinth (Lab) but not in the junctional zone (JZ), as expected. Chorionic plate (CP) mesoderm as a derivative of extra-embryonic mesoderm is EGFP-positive, as expected. Higher magnification images verify the close juxtaposition but non-overlap between EGFP, MCT1 (=SynT-I) and MCT4 (=SynT-II), indicating that *Sox2*-Flp activity is confined to fetal endothelial cells in the labyrinth which are of extra-embryonic mesoderm origin (arrow). Images are representative of n=4 placentas. **e** Verification of non-mosaic activity of *Sox2*-Flp transgenic mouse lines, assessed by using the *Ssr2* tm1a allele as a reporter, which is ubiquitously expressed throughout the entire embryo (see Suppl. Fig. 1b). Epiblast-specific expression of FLP recombinase upon male transmission of the *Sox2*-Flp transgene causes complete conversion of the LacZ-positive (blue) tm1a allele into the LacZ-negative tm1c allele in all cells of the E10.5 embryo. LacZ is also correctly lost in the fetal placental vasculature (white dotted line), whereas the blue staining in the underlying trophoblast compartment is still visible. **f** Whole-mount LacZ stainings of E12.5 embryos and placentas of *Ssr2* (tm1a/+) x *Sox2*-Flp crosses. Retention of blue staining in the junctional zone (black arrows) demonstrates that ectopic *Sox2*-Flp expression in trophoblast derivatives is minimal or absent. The orange arrow points to fetal vasculature of the yolk sac and chorionic plate mesoderm which correctly loses LacZ activity in conceptuses that carry the *Sox2*-Flp transgene. Genotypes of embryos and placentas align with those in **e**.

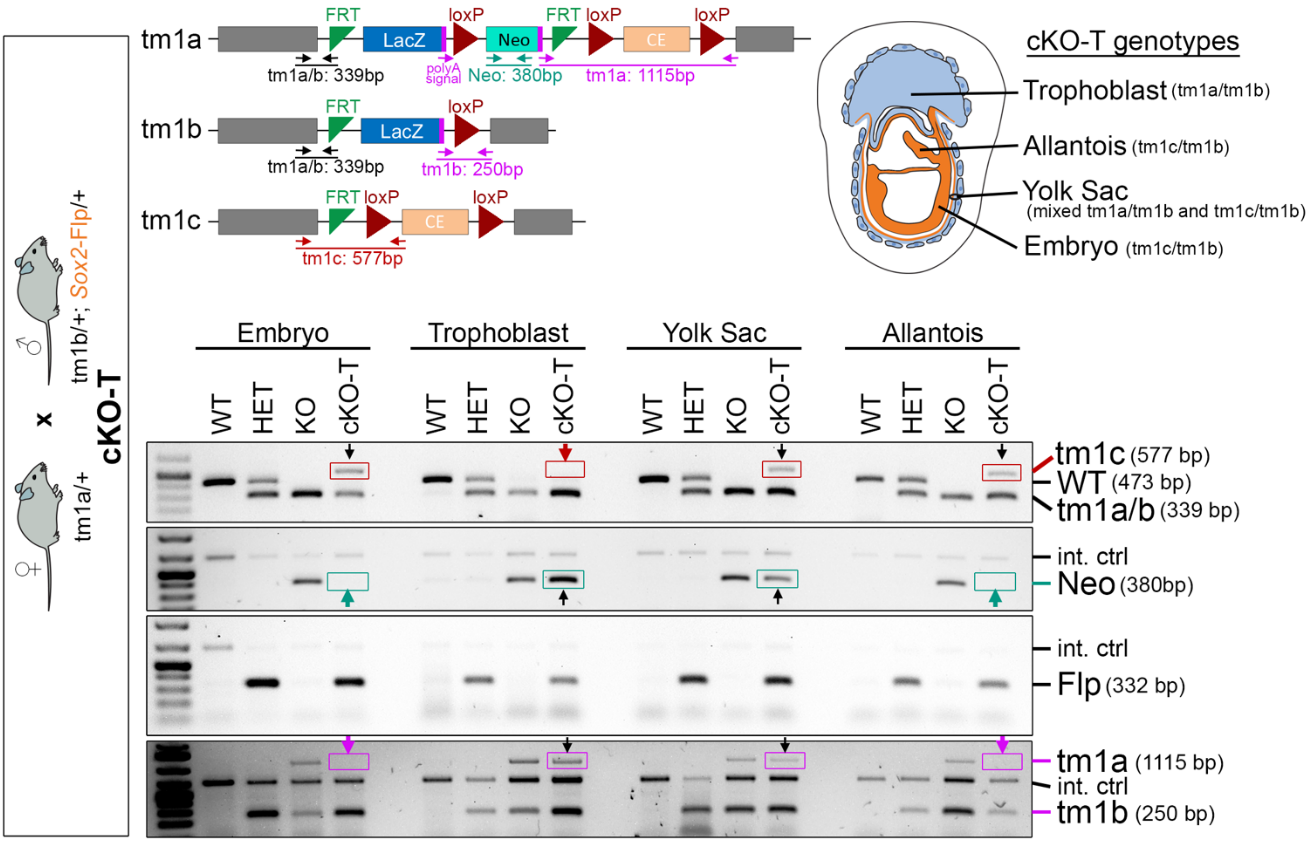

Supplementary Figure 4. Detailed genotyping to verify the lineage-specificity of *Sox2*-Flp activity.

Detailed genotyping confirmation on fine-dissected E8.5 conceptuses to verify the complete conversion of alleles mediated by *Sox2*-Flp. The cross is detailed in the box. Relevant alleles and primer locations are depicted. Tissues dissected and their lineage origin are as follows: Embryo and allantois are epiblast-derived tissues in which *Sox2*-Flp is active and consequently the tm1a allele is converted to the functional tm1c allele; Yolk Sac is of mixed lineage origin (extra-embryonic mesoderm: *Sox2*-Flp active, tm1a->tm1c; and primitive endoderm: *Sox2*-Flp inactive, tm1a); trophoblast originates from the trophectoderm and is negative for *Sox2*-Flp activity (tm1a). Conceptuses of informative genotypes were selected for PCR genotyping. Critical bands are highlighted by boxes that are colour-coded for the specific primer pair used. Coloured arrows point to the important changes in the presence or absence of bands.

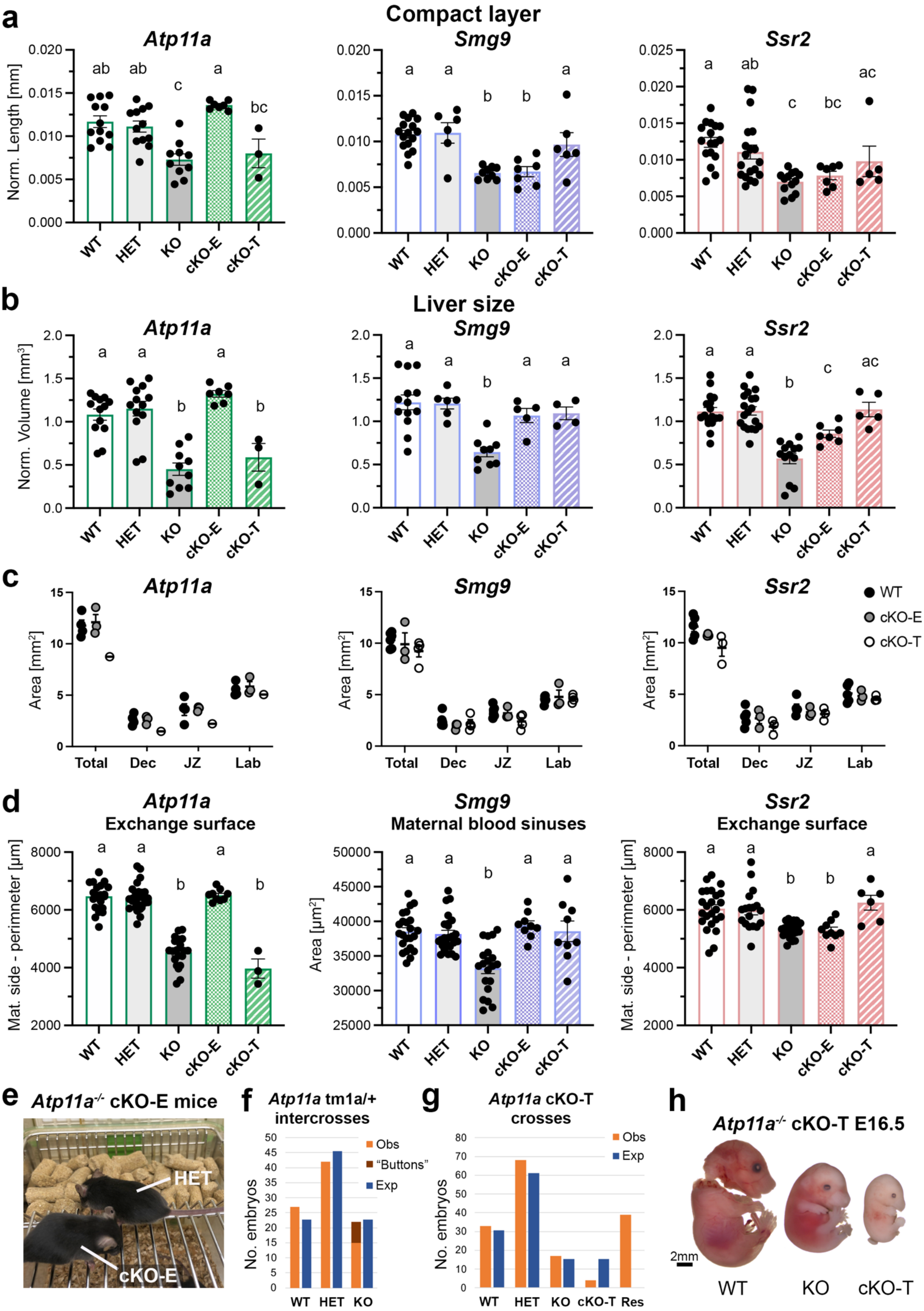

a
Compact layer
Atp11a
Smg9
Ssr2
Norm. Length [mm]
WT HET KO cKO-E cKO-T
b
Liver size
Atp11a
Smg9
Ssr2
Norm. Volume [mm³]
WT HET KO cKO-E cKO-T
c
Atp11a
Smg9
Ssr2
Area [mm²]
Total Dec JZ Lab
WT
cKO-E
cKO-T
d
Atp11a
Exchange surface
Smg9
Maternal blood sinuses
Ssr2
Exchange surface
Mat. side - perimeter [µm]
Area [µm²]
WT HET KO cKO-E cKO-T
e
Atp11a-/- cKO-E mice
HET
cKO-E
f
Atp11a tm1a/+ intercrosses
No. embryos
Obs
"Buttons"
Exp
WT HET KO
g
Atp11a cKO-T crosses
No. embryos
Obs
Exp
WT HET KO cKO-T Res
h
Atp11a-/- cKO-T E16.5
2mm
WT KO cKO-T

Supplementary Figure 5. Conditional knockout phenotypes.

a and **b** Additional quantifications of heart and liver phenotypes based on 3D µCT imaging analyses. Measurements were taken from individual control (WT/HET) and KO (KO, cKO) embryos matched across litters for *Atp11a* (WT n=12, HET n=12, KO n=10, cKO-E n=7, c-KO-T n=3), *Smg9* (WT n=16, HET n=6, KO n=9, cKO-E n=7, c-KO-T n=6), *Ssr2* (WT n=17, HET n=19, KO n=13, cKO-E n=7, c-KO-T n=5). Data are displayed as mean +/- SEM. Statistical analysis by one-way ANOVA with Holm-Šídák's multiple comparisons test. **c** Placental area measurements of WT, cKO-E and cKO-T placentas of each line, based on *Tpbpa*-demarcated decidual (Dec), junctional zone (JZ) and labyrinth (Lab) dimensions. Statistical analysis was performed using two-way ANOVA with Tukey's multiple comparisons test. Although no statistically significant differences were observed, *Atp11a*, *Smg9* and *Ssr2* cKO-T placental total areas tend to be smaller. Data are displayed as mean +/- SEM from *Atp11a* (WT n=4, cKO-E n=3, c-KO-T n=1), *Smg9* (WT n=6, cKO-E n=3, c-KO-T n=4), *Ssr2* (WT n=5, cKO-E n=3, c-KO-T n=3) placentas. **d** Analysis of placental parameters in cKO-E and cKO-T conceptuses that were fundamentally altered in the constitutive KOs. For *Atp11a* and *Ssr2*, the cKO-E and cKO-T constellation, respectively, restores placental morphology to normal, whereas the reverse cKO-T and cKO-E placentas exhibit the phenotypic alterations at full severity. The *Smg9* data reveal that the constitutive KO is a result of additive or synergistic gene function in both, the embryonic and trophoblast compartments. Placental pathology (and heart phenotype, Fig. 3) is improved in both, the cKO-E and cKO-T conditional scenarios. Data are displayed as mean +/- SEM from *Atp11a* (WT n=8, HET n=9, KO n=7, cKO-E n=3, c-KO-T n=1), *Smg9* (WT n=8, HET n=8, KO n=7, cKO-E n=3, c-KO-T n=3), *Ssr2* (WT n=8, HET n=6, KO n=8, cKO-E n=3, c-KO-T n=2) individual placentas, each measured on n=3 areas. Statistical analysis was performed with one-way ANOVA with Tukey's multiple comparisons test. **e** Picture of adult mice heterozygous and homozygous mutant for *Atp11a*, the latter a result of placental rescue in a cKO-E conceptus. *Atp11a*-null mice rescued by a functional placenta are indistinguishable from WT and HET littermates, healthy and fertile. **f** Genotype distribution of E14.5 tm1a/+ HETxHET intercrosses. About one-third of tm1a/tm1a KO conceptuses were already moribund and their placentas appeared severely bloodless and flattened, which were termed “buttons”. **g** Breeding performance and allele distribution in crosses designed to obtain *Atp11a* cKO-T conceptuses. Unlike in any of the other strains, *Atp11a*, cKO-T conceptuses were highly underrepresented at E14.5. Res = resorptions. **h** *Atp11a* WT, constitutive KO and cKO-T embryos recovered at E16.5. The cKO-T embryo is even more severely affected than the constitutive KO, and dead at this stage. This proves that *Atp11a* gene function is exclusively required in the trophoblast lineage for normal heart development and embryonic survival to term. WT and HET embryos were decapitated at this stage in compliance with animal use protocols. Source data are provided as a Source Data file. All exact *p*-values are provided in Supplementary Data 1.

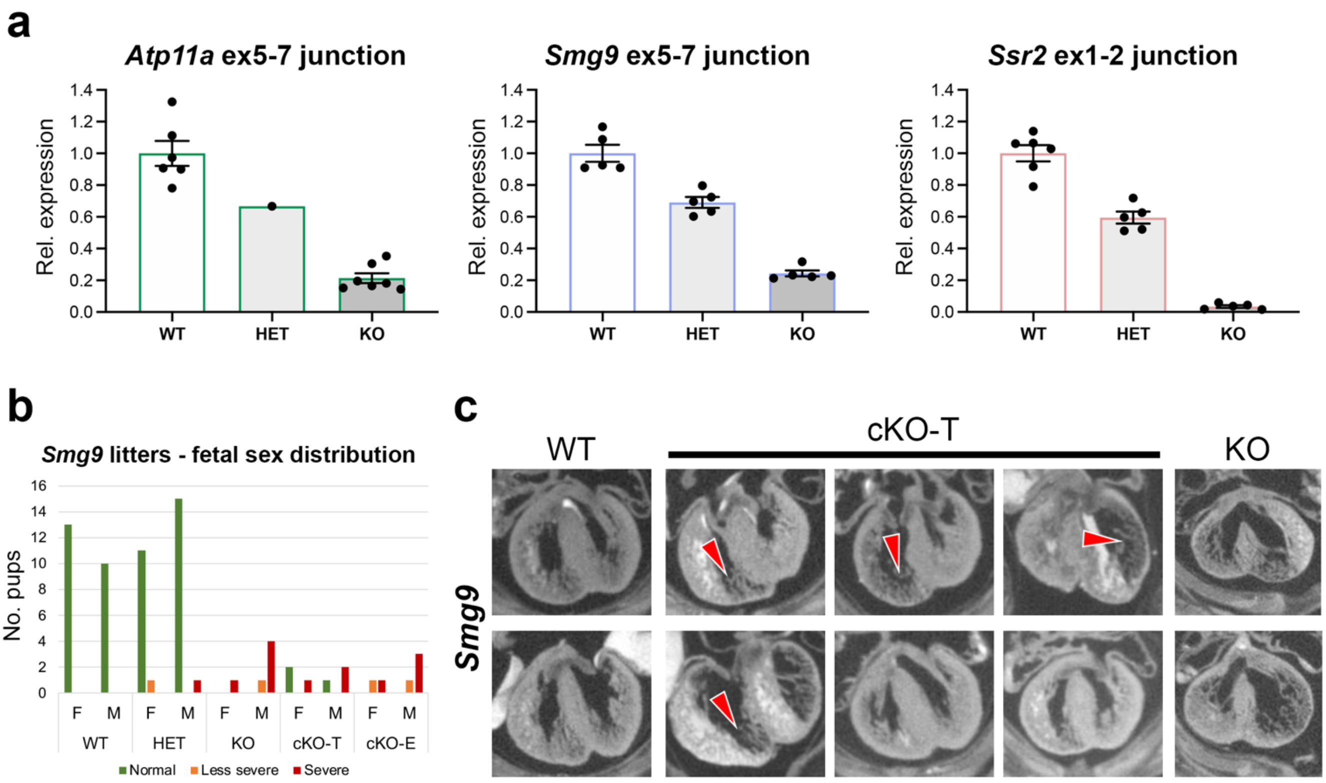

Supplementary Figure 6. Knockout alleles and additional phenotyping data for *Smg9* conditional knockouts.

a RT-qPCRs across *Atp11a* exons 5-7, *Smg9* exons 5-7 and *Ssr2* exons1-2. In the tm1a allele, these exons are separated by the LacZ-Neo cassette, precluding the amplification of a PCR product (see Supplementary Fig. 1d). Detection of ~20% product in *Atp11a* and *Smg9* KO (tm1a/tm1a) placentas indicates spurious splicing events across the cassette to produce functional *transcript*. Data are displayed as mean +/- SEM. *Atp11a*: WT n=6, HET n=1, KO n=7; Smg9: WT n=5, HET n=5, KO n=5; *Ssr2*: WT n=5, HET n=5, KO n=5. **b** Sex-specific distribution of *Smg9* conceptuses of the indicated genotypes. No sex-specific correlation of phenotype severity was observed. **c** Coronal µCT sections through the hearts of *Smg9* embryos reveal the large variability in heart pathologies of cKO-T embryos compared to the constitutive KO. Source data are provided as a Source Data file.

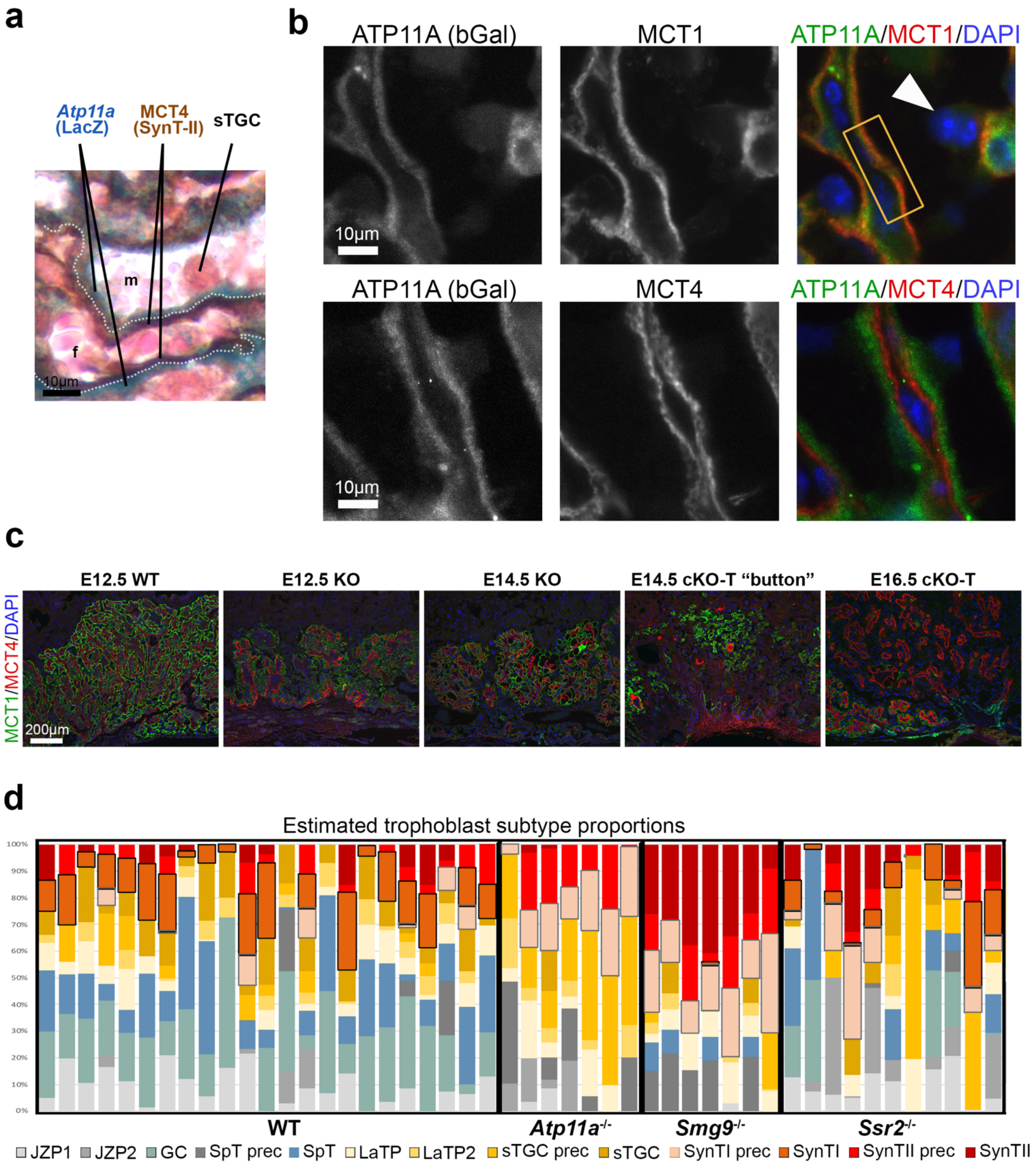

Supplementary Figure 7. Placental ATP11A expression and phenotype analysis.
a Double staining for LacZ (blue) as indicator of *Atp11a* expression and for MCT4 as marker of the syncytiotrophoblast layer-II (SynT-II) shows that ATP11A is confined to SynT-I. Staining is representative of n=6 placentas. **b** Double immunofluorescence stainings for beta-galactosidase (bGal) as indicator of ATP11A expression (green) and for MCT1 or MCT4 (red) as markers of SynT-I and SynT-II, respectively. ATP11A and MCT1 staining fully overlap in the syncytial layer, whereas ATP11A and MCT4 signals are closely juxtaposed but non-overlapping. These double stainings

demonstrate that ATP11A is confined to SynT-I. The arrowheads point to sTGC cells that are consistently negative for ATP11A expression. Stainings are representative of n=3 placentas. **c** MCT1 and MCT4 double immunofluorescence stainings on *Atp11a* placentas of the indicated genotypes recovered from E12.5 litters, and from moribund E14.5 cKO-T "buttons" (see Supplementary Fig. 5f) and E16.5 conceptuses (see Supplementary Fig. 5h). In the moribund conceptuses, the organization of the two syncytial layers that are normally closely juxtaposed is entirely lost. **d** Virtual deconvolution of placental RNA-seq data for cell type-specific composition[2,3] reveals an under-representation of mature SynT-I in all mutant placentas. Each bar represents an individual placenta, with absolute values of the proportional cell type composition being depicted. GC=glycogen cell, JZP=Junctional zone precursor, LaTP=Labyrinth precursor, SpT=spongiotrophoblast, sTGC=sinusoidal trophoblast giant cell. Source data are provided as a Source Data file.

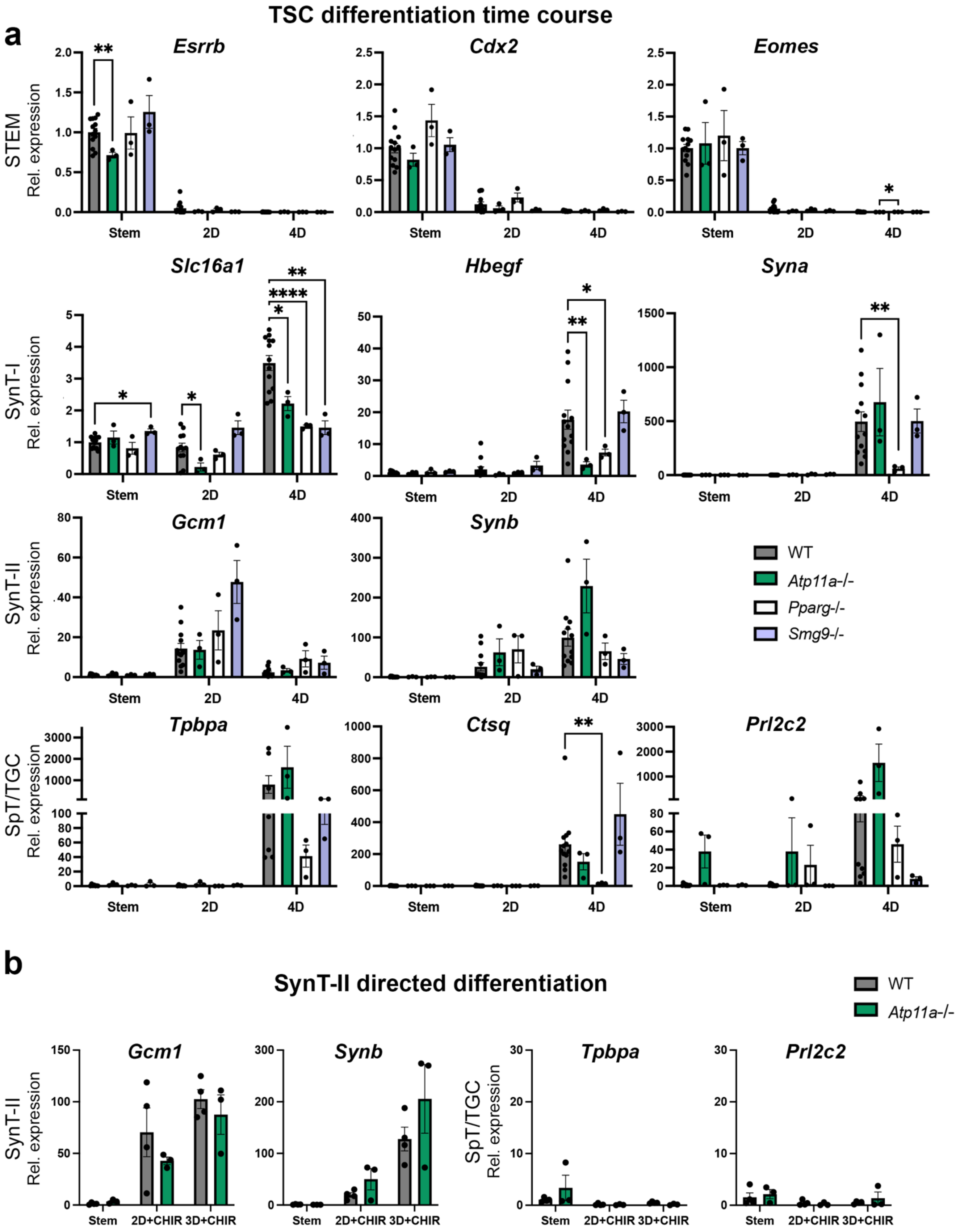
a
TSC differentiation time course
STEM
Rel. expression
Esrrb
Cdx2
Eomes
SynT-I
Slc16a1
Hbegf
Syna
SynT-II
Gcm1
Synb
SpT/TGC
Tpbpa
Ctsq
Prl2c2
Stem
2D
4D
WT
Atp11a-/-
Pparg-/-
Smg9-/-
b
SynT-II directed differentiation
2D+CHIR
3D+CHIR

Supplementary Figure 8. Trophoblast marker gene expression in KO trophoblast stem cells (TSCs).

a Trophoblast cell type-specific marker gene expression analysis across a 4-day differentiation time course by RT-qPCR in TSCs ablated for *Atp11a*, *Pparg* and *Smg9* (n=3 independent KO TSC clones for each genotype), compared to WT (n=13, except for genes *Tpbpa* (n=7), *Prl2c2* (n=10) and *Synb* (n=12)). Data are normalized to expression levels of WT cells in stem cell conditions (Stem) and displayed as mean +/- SEM. Statistical analysis was performed using two-way ANOVA and Tukey's multiple comparison test. * $p<0.05$, ** $p<0.01$. **b** Directed TSC differentiation experiment in the presence of CHIR99021, which specifically promotes SynT-II differentiation while suppressing differentiation into any other trophoblast cell type. No differences in SynT-II differentiation were observed in *Atp11a*$^{-/-}$ TSCs compared to WT controls. SpT/TGC marker genes are displayed relative to 50% expression levels at 3D standard differentiation, to visualize the comparative lack of induction of these markers. Data are mean +/- SEM of n=4 (WT) and n=3 (*Atp11a* KO) TSC clones per genotype. Statistical analysis was performed using two-way ANOVA and Tukey's multiple comparison test. SpT = spongiotrophoblast, TGC = trophoblast giant cell. Source data are provided as a Source Data file. All exact *p*-values are provided in Supplementary Data 1.

Extended TSC Differntiation Time Course

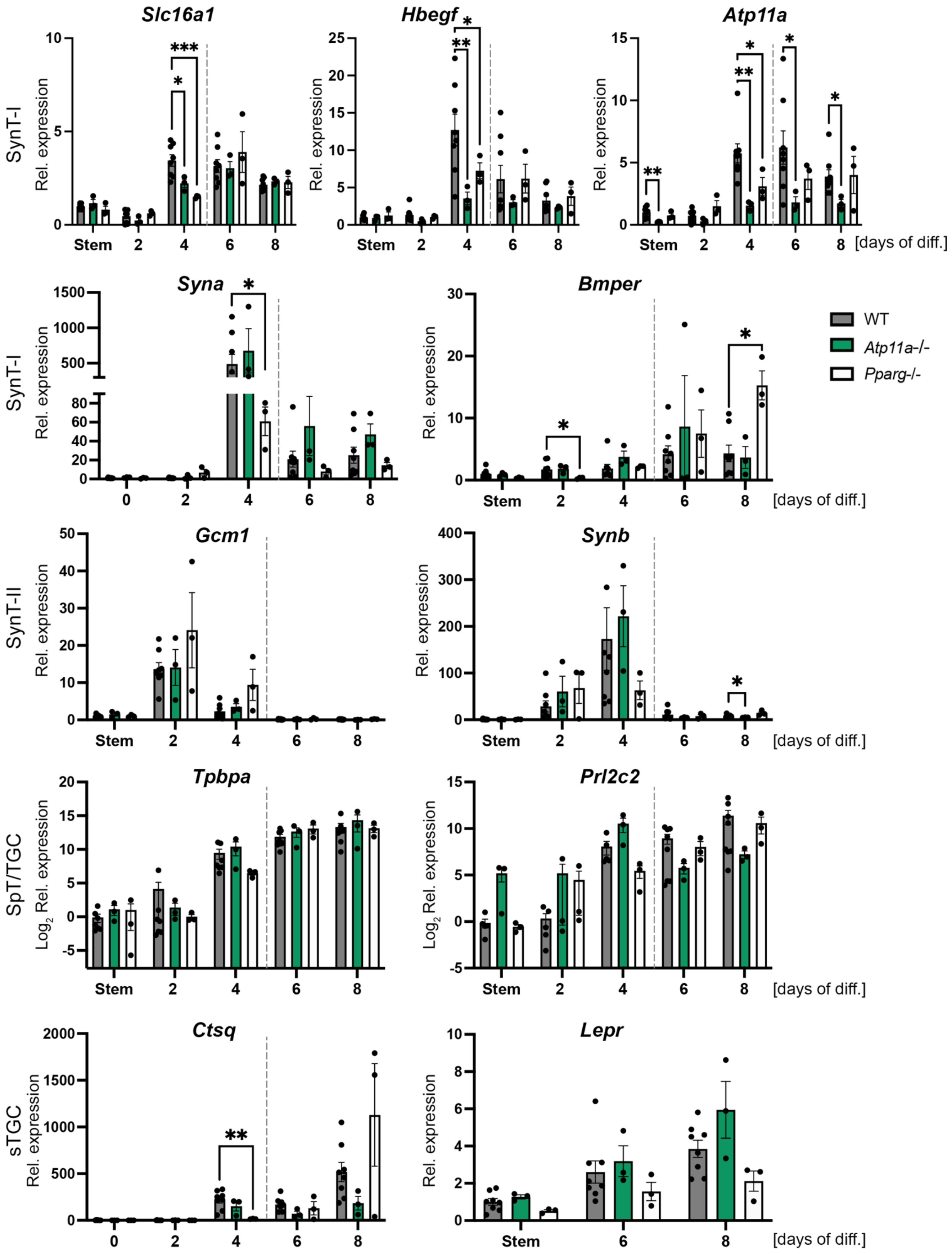

Supplementary Figure 9. Trophoblast marker gene expression in KO trophoblast stem cells (TSCs).

Trophoblast cell type-specific marker gene expression analysis across an extended 8-day (8D) differentiation time course by RT-qPCR in TSCs ablated for *Atp11a* and *Pparg*, compared to WT. Stem-to-4D differentiation conditions were designed to test for syncytialization differences; the 6D-8D time points were specifically designed to detect differences in other, non-syncytial cell types by optimizing initial plating cell numbers so not to reach confluence and not to require passaging throughout the entire time course, a procedure that always results in loss of the extremely adherent trophoblast giant cells. Thus, cell seeding numbers in 6-well plates were higher for the 2D and 4D time points (50,000 cells), and lower for the 6D (25, 000 cells) and 8D time points (15,000 cells); this difference is indicated by the dotted line. While this may obscure SynT differentiation differences at 6D and 8D (because of low cell densities during the early period that is critical for syncytial formation), the approach was designed to be specifically sensitive for detecting differentiation biases in cells of the giant cell lineage. No such differences were observed between WT and KO TSC lines. Data are normalized to expression levels of WT cells in stem cell conditions (Stem) and displayed as mean +/- SEM of WT: n=8 and KO: n=3 independent TSC clones per genotype. Statistical analysis was performed using two-way ANOVA and Tukey's multiple comparison test. * $p<0.05$, ** $p<0.01$, *** $p<0.001$. Source data are provided as a Source Data file. All exact *p*-values are provided in Supplementary Data 1.

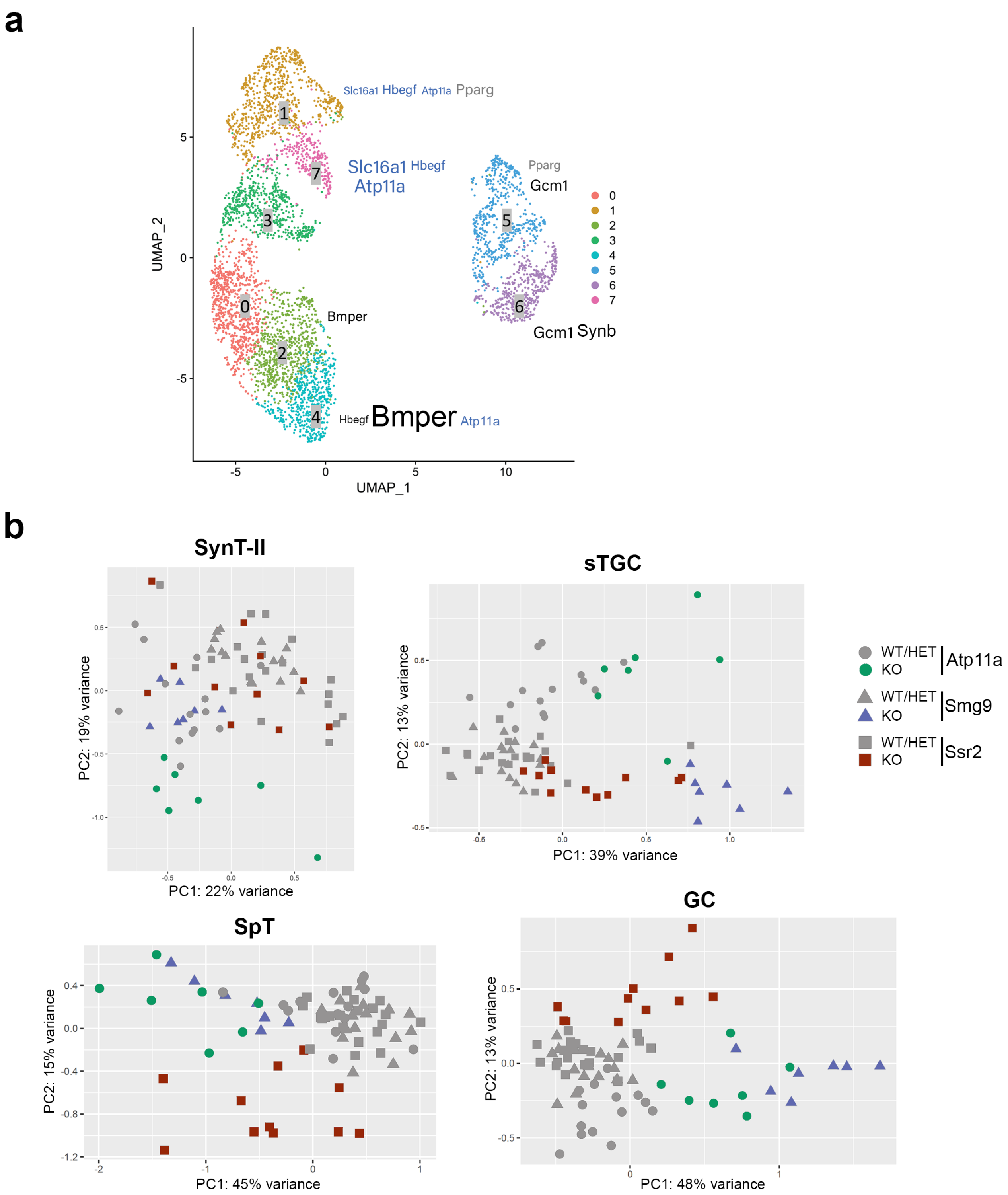

Supplementary Figure 10. Trophoblast marker gene expression in KO trophoblast stem cells (TSCs).

a UMAP plot of snRNA-seq data from Marsh and Blelloch, 2020[2] after specifically filtering on syncytiotrophoblast-specific nuclei from all developmental stages (E9.5-E14.5). SynT-I (left-hand side) and SynT-II cell nuclei cluster into two distinct groups. Moreover, SynT-I nuclei can be separated into subclusters in which subclusters 1 and 7 are demarcated by higher co-expression of *Atp11a*, *Slc16a1* (=*Mct1*), *Hbegf*, and *Pparg*. The font size of genes depicts relative expression (FC)*10. **b** Principal component analyses of bulk placental RNA-seq data filtered for SynT-II, sTGC,

spongiotrophoblast (SpT) and glycogen cell (GC) marker genes[2]. Unlike SynT-I expression patterns (Fig. 5f), none of these markers separate the KO samples as distinctly and clearly away from unaffected WT and HET samples.

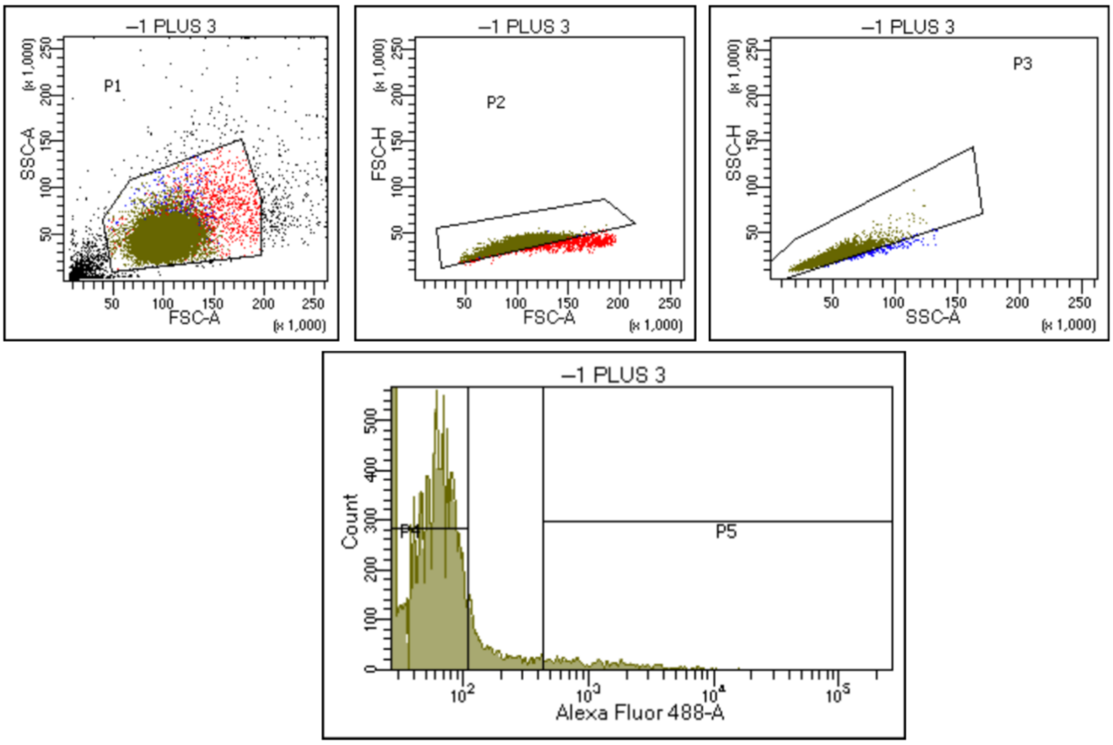

Experiment Name:	HEMBERGER-BETH-GFP-130-...
Specimen Name:	-
Tube Name:	1 PLUS 3
Record Date:	May 3, 2021 10:05:47 AM

Population	#Events	%Parent	FSC-A Mean	FSC-A Median
All Events	18,418	####	100,225	100,893
P1	16,579	90.0	103,842	101,987
P2	15,037	90.7	100,626	100,515
P3	14,644	97.4	100,323	100,311
P4	11,817	80.7	100,446	100,477
P5	1,307	8.9	97,257	96,861

Tube: 1 PLUS 3

Population	#Events	%Parent	%Total
All Events	18,418	####	100.0
P1	16,579	90.0	90.0
P2	15,037	90.7	81.6
P3	14,644	97.4	79.5
P4	11,817	80.7	64.2
P5	1,307	8.9	7.1

Supplementary Figure 11. Flow sort strategy for single cell cloning of CRISPR-Cas9 transfected TSCs.

Example of flow sorting strategy to enrich for transfected, EGFP-positive TSCs in the KO generation strategy. Population P4 = negative; Population P5 = EGFP-positive.

Supplementary Table 1: μCT measurements

Mouse Line	Measurement	Units	WT-Mean	WT-SEM	KO-Mean	KO-SEM	*p*adj
Atp11a	Embryo Length	mm	10.15	0.414	9.053	0.3257	0.0148
	Liver Volume	mm^3/mm	1.057	0.100	0.414	0.0746	<0.0001
	Ventricular Space	mm^3/mm	0.0067	0.002	0.016	0.0012	0.008
	Septal Hole	mm/mm	0	0	0.014	0.0030	0.0042
	Compact layer	mm/mm	0.0099	0.0004	0.008	0.0006	0.031
	Trabecular Layer	mm/mm	0.0280	0.0012	0.023	0.0007	0.5485
	Cardiac Muscle Volume	mm^3/mm	0.1933	0.0063	0.006	0.0128	0.1899
	Ventricle Muscle Volume	mm^3/mm	0.1214	0.004	0.141	0.0069	0.2238
	Septum Width	mm/mm	0.0405	0.0032	0.030	0.0026	0.0953
	Heart Rotation	voxels	27.30	3.081	22.15	3.469	0.2860
Smg9	Embryo Length	mm	10.89	0.279	9.474	0.1446	0.0005
	Liver Volume	mm^3/mm	1.233	0.084	0.645	0.0548	<0.0001
	Ventricular Space	mm^3/mm	0.0032	0.001	0.017	0.0029	<0.0001
	Septal Hole	mm/mm	0	0	0.020	0.0029	<0.0001
	Compact layer	mm/mm	0.0114	0.0004	0.007	0.0002	<0.0001
	Trabecular Layer	mm/mm	0.0270	0.0016	0.002	0.0025	0.9861
	Cardiac Muscle Volume	mm^3/mm	0.2227	0.0137	0.190	0.0117	0.8981
	Ventricle Muscle Volume	mm^3/mm	0.1279	0.006	0.122	0.0069	0.9840
	Septum Width	mm/mm	0.0447	0.0023	0.032	0.0027	0.0085
	Heart Rotation	voxels	36.69	2.113	54.03	6.371	0.0152
Ssr2	Embryo Length	mm	10.09	0.173	9.270	0.1864	0.0167
	Liver Volume	mm^3/mm	1.116	0.059	0.587	0.0723	<0.0001
	Ventricular Space	mm^3/mm	0.0024	0.001	0.013	0.0018	0.0005
	Septal Hole	mm/mm	0	0	0.015	0.0039	0.0003
	Compact layer	mm/mm	0.0128	0.0006	0.007	0.0003	<0.0001
	Trabecular Layer	mm/mm	0.0298	0.001	0.029	0.0018	0.9164
	Cardiac Muscle Volume	mm^3/mm	0.1931	0.0039	0.188	0.0069	0.56218
	Ventricle Muscle Volume	mm^3/mm	0.1197	0.004	0.120	0.0030	0.9997
	Septum Width	mm/mm	0.0485	0.0018	0.034	0.0025	0.0006
	Heart Rotation	voxels	30.17	3.101	21.80	4.117	0.0994

Statistical analyses were performed by one-way ANOVA with Holm-Šídák's multiple comparisons test.

Supplementary Table 2: Detailed description of heart pathologies observed in mutant embryos.

Mouse Line	ID	VSD subcategory
Atp11a-KO	1b	OA, pmVSD
	1c	OA, pmVSD
	1d	OA, pmVSD
	1e	Mild VSD/OA (or delayed closure of septum)
	3d	OA, pmVSD
	4a	OA, pmVSD
	10a	pmVSD
	12b	OA, pmVSD
Smg9-KO	2a	OA, pmVSD
	2d	DORV, IAA-B
	4f	Delayed closure of septum
	6f	OA, pmVSD
	7f	EC defect (AVCD), OA, pmVSD
	8g	OA, pmVSD
	9a	OA, pmVSD
	10d	OA, pmVSD, severe hypoplastic ventricle
	10h	OA, pmVSD
Ssr2-KO	7a	AVCD, muscular and pmVSD, PTA + IAA-B
	7b	DORV, pmVSD
	8a	DORV, IAA-B, pmVSD
	8c	DORV, pmVSD
	8g	OA, pmVSD
	12b	Delayed closure of septum
	13c	DORV, hypoplastic AscAo, pmVSD
	13f	DORV, pmVSD
	16f	DORV, pmVSD
	19d	OA, pmVSD
	20a	DORV, IAA-B, pmVSD
	20c	OA, pmVSD

AscAo = ascending aorta; AVCD = atrioventricular canal defect; DORV = double outlet right ventricle (as defined by the IPCCC description and MedGen UID 41649 as “a congenital cardiovascular malformation in which both great arteries arise entirely or predominantly from the morphologically right ventricle”); EC = endocardial cushion; IAA-B = interrupted aortic arch type B; OA = overriding aorta (as defined based on MedGen UID 120559 as “a congenital heart defect where the aorta is positioned directly over a ventricular septal defect”); pmVSD = perimembranous ventricular septal defect. “Delayed closure of septum” was assigned when there was no overt connection between the ventricles but 1-2 µCT image frames showed a very small hole in the high resolution scans. This is indicative of small pmVSDs that would be likely to close spontaneously with further gestational growth.

Supplementary Table 3: Guide RNA and primer sequences.

gRNA sequences

Target	Name	Strand	Sequence (5'-3')
Atp11a	UP	Sense	CACCGCCAGAAACCAGGTGCCGTG
		Antisense	AAACCACGGCACCTGGTTTCTGGC
	DOWN	Sense	CACCGCAGCTGGAGAGGCTCCACAC
		Antisense	AAACGTGTGGAGCCTCTCCAGCTGC
Smg9	UP	Sense	CACCGTCCACACTCACCCGCTCAGC
		Antisense	AAACGCTGAGCGGGTGAGTGTGGAC
	DOWN	Sense	CACCGGACCGTGAGTGAACCCCAC
		Antisense	AAACGTGGGGTTCACTCACGGTCCC
Pparg	UP	Sense	CACCGTACTGTCTCAGAAGAGGCGC
		Antisense	AAACGCGCCTCTTCTGAGACAGTAC
	DOWN1	Sense	CACCGCATCTCAGTGGATCTTGCAG
		Antisense	AAACCTGCAAGATCCACTGAGATGC
	DOWN2	Sense	CACCGATATTACAGCATTAGTCCAT
		Antisense	AAACATGGACTAATGCTGTAATATC

Table of primers

Experiment	Target	Primer	Sequence (5'-3')
**Genotyping Mice**	Atp11a-tm1a/1c/1d	FWD	CACGTCTGTGTTCCTGTGTCC
		REV	TATTTGATGCACCTGCCCTG
		Cas1-R	TCGTGGTATCGTTATGCGCC
	Ssr2-tm1a/1c/1d	FWD	AGGCAGGGATAAATTCAGCTTG
		REV	TGACTGGAAAATCCACCTGC
		Cas1-R	TCGTGGTATCGTTATGCGCC
	Smg9-tm1a/1c/1d	FWD	TGCCCCTCTCACCTCTTAGC
		REV	ACCTGAGACCTTTGCCTTGG
		Cas1-R	TCGTGGTATCGTTATGCGCC
	tm1b-generic	FWD	CTCCCACACCTCCCCCTGAA
		REV	TGAACTGATGGCGAGCTCAGA
	Flp	FWD	TCTTTAGCGCAAGGGGTAGGATCG
		REV	GTCCTGGCCACGGCAGAAGC
	Cre	FWD	CAATTTACTGACCGTACACC
		REV	TCCCCAGAAATGCCAGATTAC
	Cts8-internal control	FWD	CAGTTTGGATTCTGAATGGC
		REV	ACAGCCTCTTTTTCTCCAGTC
	Neomycin	FWD	TGCTCCTGCCGAGAAAGTATCCATCATGGC
		REV	CGCCAAGCTCTTCAGCAATATCACGGGTAG
	Sry	FWD	TTGTCTAGAGAGCATGGAGGGCCATGTCAA
		REV	CCACTCCTCTGTGACACTTTAGCCCTCCGA
**RT-PCR**	Essrb	FWD	AGTACAAGCGACGGCTGG
		REV	CCTAGTAGATTCGAGACGATCTTAGTCA
	Eomes	FWD	TCGCTGTGACGGCCTACCAA
		REV	AGGGGAATCCGTGGGAGATGGA

	Cdx2	FWD	AGTGAGCTGGCTGCCACACT
		REV	GCTGCTGCTGCTTCTTCTTGA
	Slc16a1 (Mct1)	FWD	CTCCAGTGCTGTGGGCTTGG
		REV	GCGATGATGAGGATCACGCCA
	Hbegf	FWD	GTGCTCAGGGGGTCCAGGACTT
		REV	TTCTTTGCTTGGGGTGGCCAGG
	Atp11a	FWD	ACTGGACGTTTCTCGGCGTT
		REV	TGAACACCAGCGTCCCGAAG
	Bmper	FWD	CAGAGAGGCGCCTGCTGTGAAC
		REV	AGCCGGGGTTTGCCACTTGAAG
	Syna	FWD	CCTCACCTCCCAGGCCCCTC
		REV	GGCAGGGAGTTTGCCCACGA
	Gcm1	FWD	ACTTCTGGAGGCACGACGGA
		REV	TCGGGATTTCAGCAGGAAGCG
	Synb	FWD	GGCAACTCTCCGCAGCTGACAC
		REV	ACGAACCAGCTGTGCTTGAGCC
	Ctsq	FWD	AATTGGCTATGGTTATGTGGGA
		REV	TCACACAGTAGGGTATTGGG
	Lepr	FWD	GCTGGGATGTGCGTTGGAGGAC
		REV	TGCTGGTCGCGTCGGAGTCATA
	Tpbpa	FWD	ACTGGAGTGCCCAGCACAGC
		REV	GCAGTTCAGCATCCAACTGCG
	Plf/Prl2c2	FWD	AACGCAGTCCGGAACGGGG
		REV	TGTCTAGGCAGCTGATCATGCCA
	Sdha	FWD	TGGTGAGAACAAGAAGGCATCA
		REV	CGCCTACAACCACAGCATCA
	Atp11a ex5-7	FWD	GTCGGAAGCTGAGGGTTGGG
		REV	GTGGCGTGCAAGCTGTCAAC
	Smg9 ex5-7	FWD	AGAGCGCATGAAGCACAGCA
		REV	GGTTGCCCCCTCGTTCCTTC
	Ssr2 ex1-2	FWD	CATGCTTGGGTCGATTGCTCAC
		REV	ATCTAATGCAGCGCTGGAGCC
**Genotyping CRISPR in TSCs**	Smg9-ex4-out	FWD	GCTGCGTTCCCTCTCCTCAGGA
		REV	GTTCTGGAGGGGTGTGGACGGA
	Smg9-ex4-in 3' end	FWD	GCTGCGTTCCCTCTCCTCAGGA
		REV	GTGTGGGTCTCTGCCCCTCCTT
	Smg9-WT	FWD	TGCCCCTCTCACCTCTTAGC
		REV	ACCTGAGACCTTTGCCTTGG
	Pparg_out	FWD	TGGGGTGTTGTGACTTGCCTGA
		REV	TGACCCCATGCCTTTGATGCACA
	Pparg_in 3' end	FWD	GAAAGCAGCGGGAAGTTTGG
		REV	GAGGCCTGTTGTAGAGCTGG
	Atp11a_out	FWD	GGATGCACTTAGTTCAGAGTTGA
		REV	CTGTGAGGGTGCTGCTCA
	Atp11a_in 3' end	FWD	GGAAGAGCAGGATGGCTAAGTCACC
		REV	GACTTCATAGCCTGTGAGGGTGCTG

Raw images – Genotyping gels of Suppl. Fig. 3e

Lane loading as indicated in Suppl. Fig. 3e

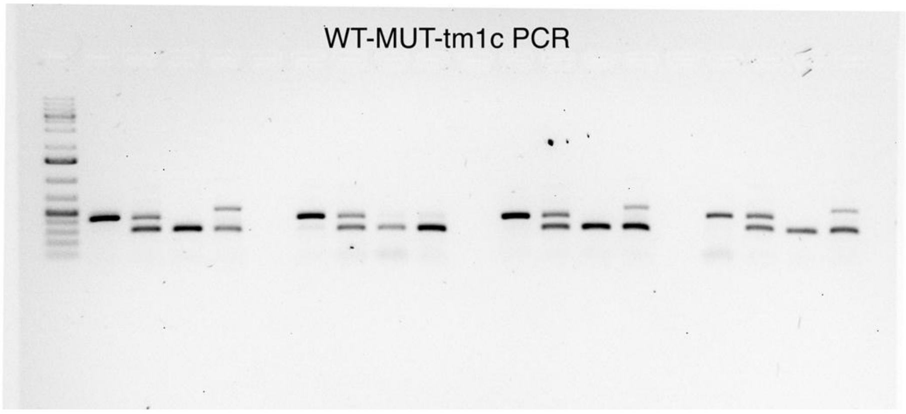

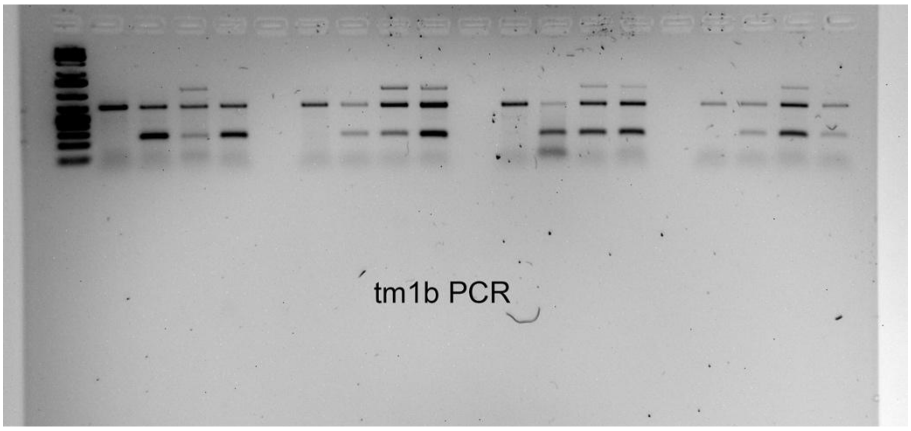

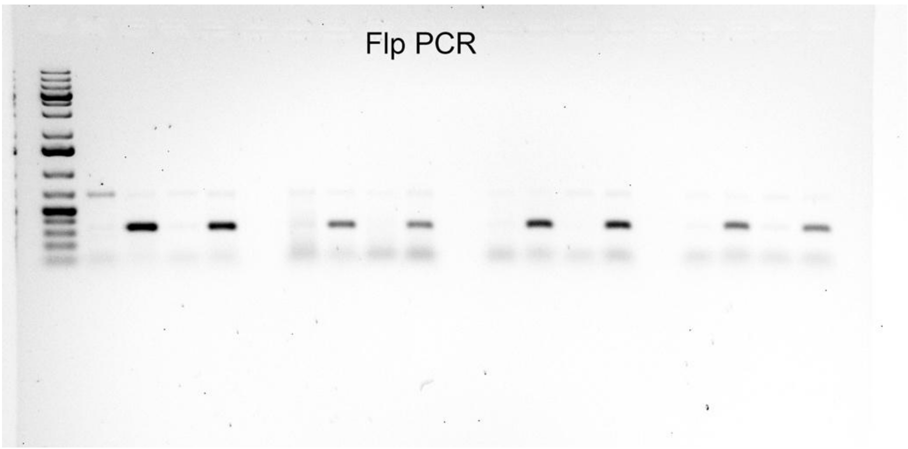

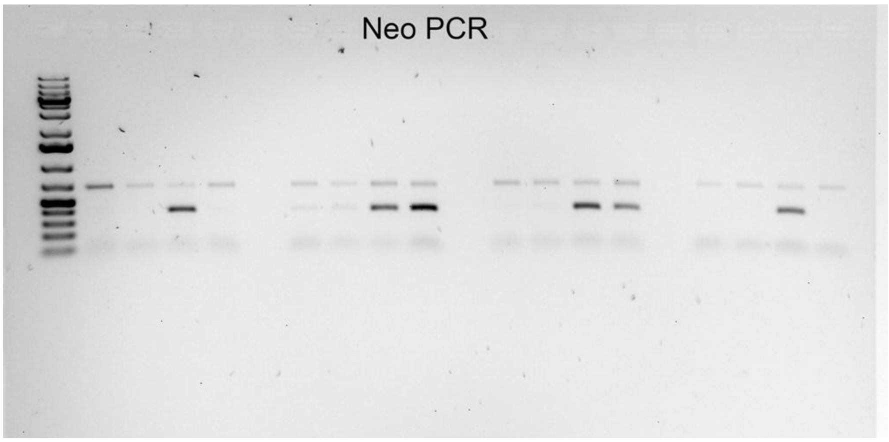

Supplemental References

1. He, P., Williams, B. A., Trout, D., Marinov, G. K., Amrhein, H., Berghella, L. *et al.* The changing mouse embryo transcriptome at whole tissue and single-cell resolution. *Nature* **583**, 760-767 (2020).
2. Marsh, B. & Blelloch, R. Single nuclei RNA-seq of mouse placental labyrinth development. *Elife* **9** (2020).
3. Jew, B., Alvarez, M., Rahmani, E., Miao, Z., Ko, A., Garske, K. M. *et al.* Accurate estimation of cell composition in bulk expression through robust integration of single-cell information. *Nat. Commun.* **11**, 1971 (2020).

Supplementary Note

Detailed description of μCT imaging and image analysis

Embryos were fixed overnight in 4% PFA and stored in PBS. Fixed embryos were then stained overnight in Lugol's iodine (2.5% w/v I_2KI) dye and embed in 1% agarose immediately before scanning. Images were obtained on a ZEISS Xradia Versa 520 X-ray microscope 186 (Carl Zeiss AG, Oberkochen, Germany) with the following settings: 0.4X objective, 50kV, 4W, Binning 2, exposure: 2s. The resolution obtained was 10-12.89μm/pixel. Reconstruction was completed by the the ZEISS XMReconstructor software and converted to TIFF files with ZEISS XMcontroller software. Segmentation of images obtained were completed in Slicer (version 4.11.20210226) with the MarkupsTOModel and SegmentEditorExtraEffects plugins. The following volumes were collected:

Volume of Ventricular Space:

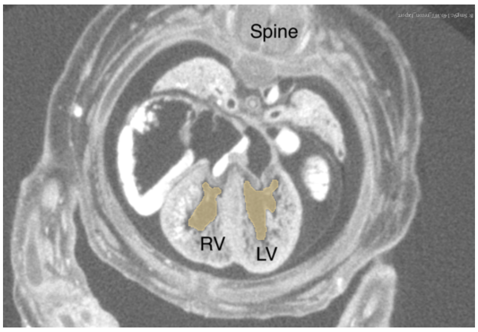

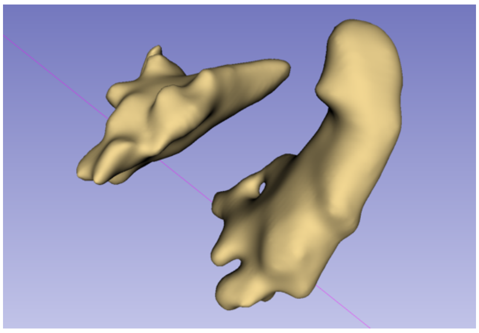

2D- No VSD 3D – No VSD

Volume of Ventricular muscle:

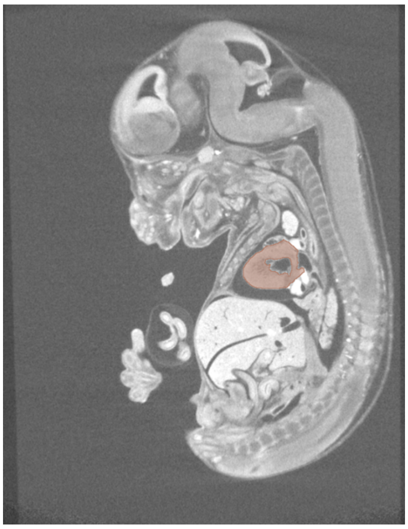

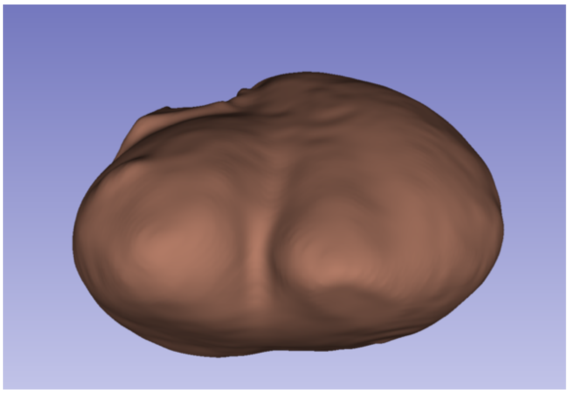

2D 3D

Volume of Liver:

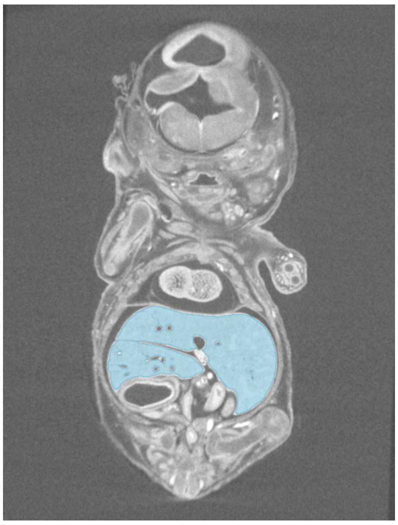

2D

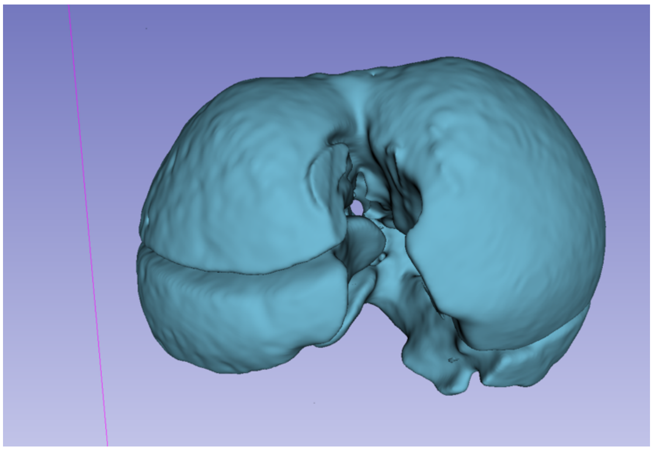

3D

Septal Hole Size:

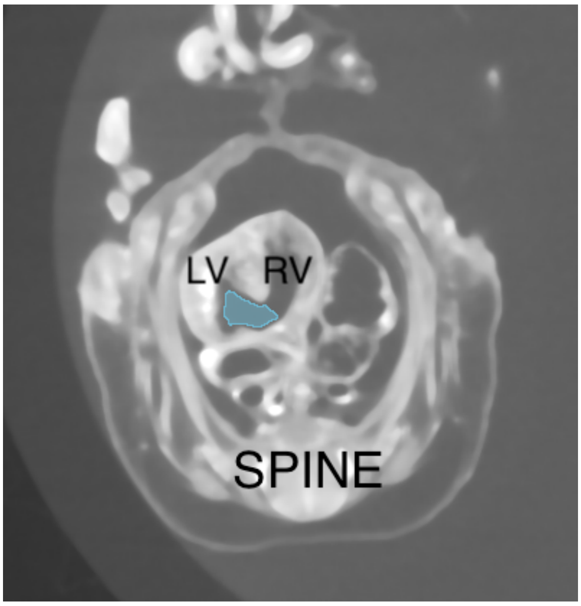

2D

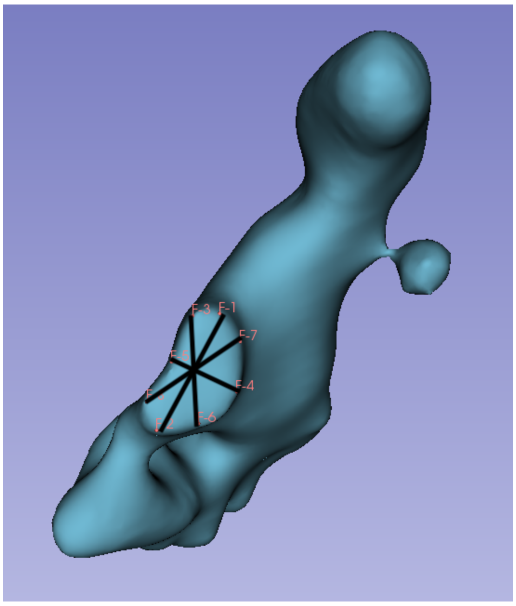

3D

Average of the 4 lengths from the cross-section of the septal hole.

Ventricular Thickness (1), Compact Layer Thickness and Trabecular Layer Thickness:

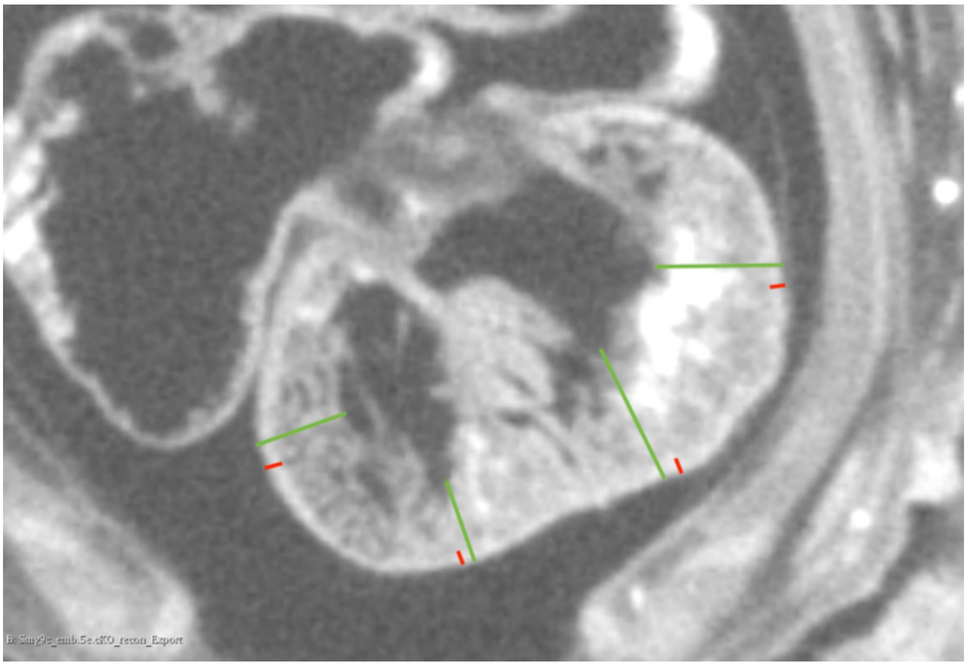

Ventricular Thickness = Average of lengths of green lines
Compact layer Thickness = Average of lengths of red lines
Trabecular Layer Thickness = Average of green line lengths - Average of red line lengths

Examples of perimembranous and muscular VSDs (flood-filled ventricular spaces- see "Volume of Ventricular Space"):

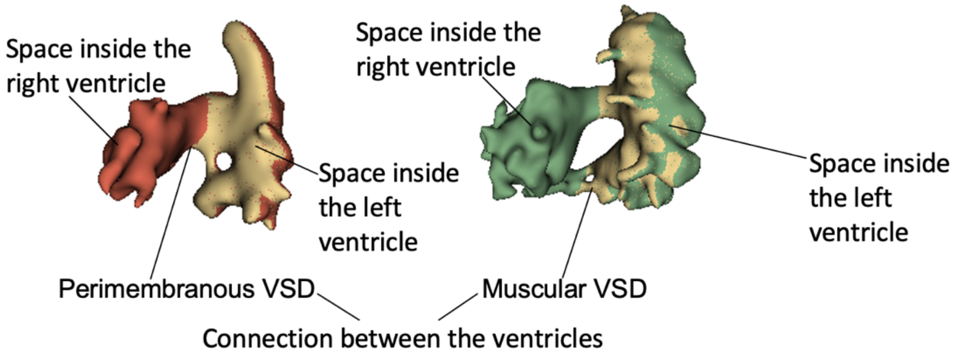

Embryo Length:

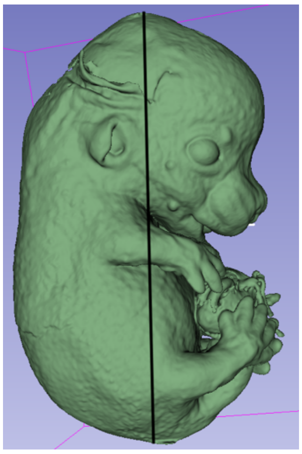

Measurements were then normalized to the embryo length to account for differences in embryo size. 2D and 3D measurements were normalized by embryo length and the means were compared in GraphPad (9.4) with a one-way ANOVA and Holm-Šídák's post-hoc test.
