# Supplementary material for: Defects in placental syncytiotrophoblast cells are a common cause of developmental heart disease
Source: Nat Commun. 2023 Mar 1;14:1174. doi: 10.1038/s41467-023-36740-5 (PMC9978031; doi:10.1038/s41467-023-36740-5)
Supplement: Supplementary file 2 — Description of Additional Supplementary Files [file 41467_2023_36740_MOESM2_ESM.pdf]

Description of Additional Supplementary Files

File Name: Supplementary Data 1
Description: Listing *p*-values for all experimental comparisons performed.

File Name: Supplementary Movie 1
Description: Movie of reconstructed μCT image stacks through a normal E14.5 mouse heart.

File Name: Supplementary Movie 2
Description: Movie of reconstructed μCT image stacks through a E14.5 mouse heart displaying a perimembranous ventricular septal defect.

File Name: Supplementary Movie 3
Description: Movie of reconstructed μCT image stacks through a E14.5 mouse heart displaying a muscular ventricular septal defect.
