# Supplementary material for: Defects in placental syncytiotrophoblast cells are a common cause of developmental heart disease
Source: Nat Commun. 2023 Mar 1;14:1174. doi: 10.1038/s41467-023-36740-5 (PMC9978031; doi:10.1038/s41467-023-36740-5)
Supplement: Supplementary file 7 — Reporting Summary [file 41467_2023_36740_MOESM7_ESM.pdf]

nature portfolio

Corresponding author(s): Dr Myriam Hemberger and Dr Wendy Dean

Last updated by author(s): Feb 3, 2023

Reporting Summary

Nature Portfolio wishes to improve the reproducibility of the work that we publish. This form provides structure for consistency and transparency in reporting. For further information on Nature Portfolio policies, see our Editorial Policies and the Editorial Policy Checklist.

Statistics

For all statistical analyses, confirm that the following items are present in the figure legend, table legend, main text, or Methods section.

n/a	Confirmed	
☐	☒	The exact sample size (*n*) for each experimental group/condition, given as a discrete number and unit of measurement
☐	☒	A statement on whether measurements were taken from distinct samples or whether the same sample was measured repeatedly
☐	☒	The statistical test(s) used AND whether they are one- or two-sided<br>*Only common tests should be described solely by name; describe more complex techniques in the Methods section.*
☐	☒	A description of all covariates tested
☐	☒	A description of any assumptions or corrections, such as tests of normality and adjustment for multiple comparisons
☐	☒	A full description of the statistical parameters including central tendency (e.g. means) or other basic estimates (e.g. regression coefficient) AND variation (e.g. standard deviation) or associated estimates of uncertainty (e.g. confidence intervals)
☐	☒	For null hypothesis testing, the test statistic (e.g. *F*, *t*, *r*) with confidence intervals, effect sizes, degrees of freedom and *P* value noted<br>*Give P values as exact values whenever suitable.*
☒	☐	For Bayesian analysis, information on the choice of priors and Markov chain Monte Carlo settings
☒	☐	For hierarchical and complex designs, identification of the appropriate level for tests and full reporting of outcomes
☒	☐	Estimates of effect sizes (e.g. Cohen's *d*, Pearson's *r*), indicating how they were calculated

Our web collection on statistics for biologists contains articles on many of the points above.

Software and code

Policy information about availability of computer code

Data collection	Micro-CT scans were reconstructed with ZEISS XMReconstructor software then converted to a TIFF using ZEISS XMcontroller. Image analysis was conducted using Slicer software (version 4.11.20210226). All RT-PCR plates in this study were read on the CFX384 (Bio-Rad) and Bio-Rad CFX Maestro Software.
Data analysis	Micro-CT image segmentation and quantification was completed with Slicer (version 4.11.20210226). Maternal and fetal blood space parameters in the placenta were analyzed in ImageJ (version 1.53). All RNAseq data were aligned with STAR (2.6.1a_08-27) and count tables were generated with HT-Seq (bioconda 2018.11). Count tables were analyzed in the R console (version 3.4.3 and 4.1.0) along with the following packages: DESeq2 (1.32.0), EdgeR (3.34.1), ggplot2 (3.3.5), gplots (3.1.1), BiomaRt (2.48.3), Rcolorbrewer (1.1-2), limma (3.48.3), VennDiagram (1.7.0), BisqueRNA (1.0.5), devtools (2.4.2), loomR (0.2.1.9000), stringr (1.4.0). Statistical analyses for all data, except RNAseq, was performed in Graph Pad Prism (version 9.4.1). Website analysis tools used were Webgestalt (http://www.webgestalt.org/) and Metascape (https://metascape.org/).<br>Flow cytometry-based sorting of EGFP-positive TSCs was performed using BD FACSDiva Software (8.0.1 build 2014 07 03 11 47) and Firmware (version 1.3).

For manuscripts utilizing custom algorithms or software that are central to the research but not yet described in published literature, software must be made available to editors and reviewers. We strongly encourage code deposition in a community repository (e.g. GitHub). See the Nature Portfolio guidelines for submitting code & software for further information.

Data

Policy information about availability of data

All manuscripts must include a data availability statement. This statement should provide the following information, where applicable:

- Accession codes, unique identifiers, or web links for publicly available datasets
- A description of any restrictions on data availability
- For clinical datasets or third party data, please ensure that the statement adheres to our policy

Placental mRNA sequencing data is available on GEO accession GSE204859 (https://www.ncbi.nlm.nih.gov/geo/query/acc.cgi?acc=GSE204859) and the mouse genome reference was downloaded from ensembl FTP downloads (https://uswest.ensembl.org/info/data/ftp/index.html). A source data file containing every data point analyzed and plotted is also included.

Human research participants

Policy information about studies involving human research participants and Sex and Gender in Research.

Reporting on sex and gender	NA
Population characteristics	NA
Recruitment	NA
Ethics oversight	NA

Note that full information on the approval of the study protocol must also be provided in the manuscript.

Field-specific reporting

Please select the one below that is the best fit for your research. If you are not sure, read the appropriate sections before making your selection.

☒ Life sciences ☐ Behavioural & social sciences ☐ Ecological, evolutionary & environmental sciences

For a reference copy of the document with all sections, see nature.com/documents/nr-reporting-summary-flat.pdf

Life sciences study design

All studies must disclose on these points even when the disclosure is negative.

Sample size

Sample sizes for knockout embryos was determined based on previous data in which an n=3 revealed phenotypes that occurred with full penetrance but variable expressivity (doi: 10.1038/nature26002). For the current study, we chose a sample size of n>=8 for all tm1a heterozygous intercrosses (micro-CT scanning, placental mRNA sequencing/histology) and n>=6 for conditional knock-outs (micro-CT scanning, placental histology) for each genotype in all of the three mouse mutant lines to reliably capture any phenoytpe variations. The exception was Atp11a-cKO-T samples, where only 4 E14.5 conceptuses were obtained, of which 3 embryos could be imaged by micro-CT scanning (the fourth embryo was dead). As shown in our manuscript, this line was subviable at E14.5. All data points are plotted and/or the exact numbers of samples that went into graphs are provided.

Based on our extensive experience with mouse KO TSC lines and considering the necessity to perform qPCR reactions within the same microwell plate for all genes analysed, at least three independent KO clones and four independent wild-type clones that were derived in parallel to each KO from the exact same starting TSC population, were obtained from every CRISPR-Cas9 KO experiment targetting Smg9, Atp11a, and Pparg, . The proliferation assay throughout differentiation was repeated 3 independent times for the four mutant TSC lines and the corresponding wild-types. RT-qPCRs for trophoblast stem cell and differentiation markers were run in triplicate on samples from 5 time-points for three Atp11a, Smg9 and Pparg KOs, as well as the corresponding four wild-type clones that were co-derived in parallel to the KO clones from the same parental TSC population. Due to the volumes of values measured any additional clones from the transfections were frozen and not analyzed in this study.

Data exclusions

No data was excluded from the study

Replication

For all experiments in mice, at least one control and mutant sample was collected per litter to achieve both intra-litter and inter-litter controls and thereby the highest statistical power. Approximately equal numbers of males and females were sampled.

Growth of KO TSCs relative to wild-type clones in both stem and differentiation conditions were assessed three independent times. Values are represented as an average of the technical replicates. The RT-qPCR data was collected once, however, Atp11a and Smg9 KO clones were repeated for the extended time course and markers demonstrated similar trends. For the extended time course, the data shown have been integrated between the short-term and long-term time course for consistency.

	In all settings, replicate experiments were consistent in their principle outcome.
Randomization	Micro-CT samples (i.e., embryos) were scanned and segmented in batches with random genotypes. Histological slides of placentas always included matched genotypes per slide, i.e. KO and WT samples embedded and treated in parallel. Analyses of placental vascular parameters were performed in a blinded fashion (i.e. the genotype was not known to the person performing the analysis while performing these measurements). For TSC culture, wild-type and KO clones were always grown and differentiated in parallel.
Blinding	Analyses of placental vascular parameters were performed in a blinded fashion. Measurements of TSC fusion indices of wild-type and Atp11a KO cells were also conducted in a blinded manner. For micro-CT analyses, the data were not blinded as the entire methodology was based on computational quantifications and no bias could have possibly been introduced. The same holds true for the anlaysis of RT-qPCR data from wild-type and KO TSCs.

Reporting for specific materials, systems and methods

We require information from authors about some types of materials, experimental systems and methods used in many studies. Here, indicate whether each material, system or method listed is relevant to your study. If you are not sure if a list item applies to your research, read the appropriate section before selecting a response.

Materials & experimental systems

n/a	Involved in the study
☐	☒ Antibodies
☐	☒ Eukaryotic cell lines
☒	☐ Palaeontology and archaeology
☐	☒ Animals and other organisms
☒	☐ Clinical data
☒	☐ Dual use research of concern

Methods

n/a	Involved in the study
☒	☐ ChIP-seq
☐	☒ Flow cytometry
☒	☐ MRI-based neuroimaging

Antibodies

Antibodies used	MCT1 (1:200, Sigma AB1286-I, lot 3824852)<br>MCT4 (1:200, Sigma AB3314P, lot 3824828)<br>RXRa (1:100, Abcam ab125001, lot GR3197430-9)<br>phospho-histone H3S10 (1:200, Millipore-Sigma #06-570, lot 3869133).<br>beta-galactosidase (1:200, Cell Signaling #27198)<br>beta-galactosidase (1:200, Abcam ab9361)<br>AlexaFluor488 and 568 secondary antibodies (1:500, ThermoFisher A-21202, A-10037, A-21206, A-10042, A-11041, A-11039)<br>Horseradish peroxidase conjugated anti-rabbit secondary antibody (1:200, Cell Signalling 7074S)
Validation	MCT1 and MCT4 staining patterns are well-established and are localized to SynT-I and SynT-II, respectively. The antibodies were verified in double IF staining experiments to recapitulate this exact staining pattern on mouse placentas (see Fig. 5, Suppl. Figs. 3 and 7) . Cell type-specific staining was also ascertained by double-IF on mouse placentas which shows juxtaposition but non-overlap of staining patterns in the placental labyrinth, as expected. RXRa is known to be localized to murine sinusoidal trophoblast giant cells in the placental labyrinth, and this expected pattern was validated by co-measurements of nuclear sizes of positive and negative cells. PHH3 is one of the best-known proliferative markers; antibody validation was performed on cultured mouse TSCs in which the positivity of metaphase chromosomes is evident. beta-galactosidase antibodies are also well-established and were validated on LacZ-positive versus LacZ-negative specimens, the latter serving as negative control. Moreover, in all stainings tissue negative for LacZ were included alongside tissue that carried a LacZ transgene (or knock-in), thus providing a control for the specificity of staining patterns. All secondary antibodies were verified in no-primary antibody control staining experiments that produced no signals.

Eukaryotic cell lines

Policy information about cell lines and Sex and Gender in Research

Cell line source(s)	The TS-Rs26 trophoblast stem cell line was kindly provided by the Rossant Lab (Toronto, Canada).
Authentication	TSCs express markers distinct in their combination for trophoblast stem cells, and are capable of differentiating into syntytiotrophoblast and trophoblast giant cells, again cell types that are unique to the trophoblast lineage.
Mycoplasma contamination	Upon arrival to the lab and prior to knock-out generation via CRISPR, stock TS-Rs26 cells were tested for mycoplasma using a PCR kit (e-Myco, Cat. No 25233, iNtRON Biotechnology DR). All stocks were negative.
Commonly misidentified lines<br>(See ICLAC register)	No commonly misidentified cell lines were used.

Animals and other research organisms

Policy information about studies involving animals; ARRIVE guidelines recommended for reporting animal research, and Sex and Gender in Research

Laboratory animals	Sperm carrying the targetted alleles Atp11a tm1a(KOMP)Wtsi, Smg9 tm1a(EUCOMM)Wtsi and Ssr2 tm1a(EUCOMM)Wtsi were purchased from the International Mouse Phenotyping Consortium (IMPC) and used for in vitro fertilization of C57BL/6N (Charles River) oocytes to re-derive the corresponding mouse strains. All strains were maintained as heterozygotes by routine breeding to C57BL/6N-Elite (Charles River) mice. For conversion of tm1a into tm1b, tm1c and tm1d alleles, we used the Pgk1-FlpO line (B6.Cg-Tg(Pgk1-flpo)10Sykr/J, strain ID 011065) and the Sox2-Cre line (B6.Cg-Edil3<Tg(Sox2-cre)1Amc>/J, strain ID 008454) as appropriate, which we purchased from Jackson Laboratories. Sox2-FlpO transgenic males were crossed to the RCE:FRT reporter strain (MMRRC Strain #032038-JAX) which harbours the R26R CAG-boosted EGFP (RCE) reporter allele with a FRT-flanked STOP cassette upstream of an enhanced green fluorescent protein (EGFP) gene. Female mice used were between 8-30 weeks of age, male mice were between 8-52 weeks of age. All mice were housed in IVC cages with 12 hour light-dark cycles under ambient temperature (~22ºC) and humidity conditions.
Wild animals	No wild animals were used in this study.
Reporting on sex	Samples for all genotypes included both male and female mice. While sex was not a primary focus of this study, sample variation due to sex was assessed and figures are included in the supplement. Placental RNAseq data did not cluster on PCAs based on sex. This suggests that overall there was no significant influence of sex on the phenotypes observed. Additionally, sex of the embryo did not predict the severity of the heart defect, as demonstrated in the supplementary figure of Smg9 frequency for each severity category.
Field-collected samples	No field-collected samples were used in this study.
Ethics oversight	All animal work was conducted with approval by the University of Calgary's animal care committee, and with appropriate Health Sciences Animal Care Committee (HSACC)-approved animal use protocols in place (protocol number AC18-0191, renewal protocol AC22-0147).

Note that full information on the approval of the study protocol must also be provided in the manuscript.

Flow Cytometry

Plots

Confirm that:

☒ The axis labels state the marker and fluorochrome used (e.g. CD4-FITC).

☒ The axis scales are clearly visible. Include numbers along axes only for bottom left plot of group (a 'group' is an analysis of identical markers).

☒ All plots are contour plots with outliers or pseudocolor plots.

☒ A numerical value for number of cells or percentage (with statistics) is provided.

Methodology

Sample preparation	Flow cytometry was only used to single cell-sort trophoblast stem cells (TSCs) based on EGFP positivity. It was NOT used to determine quantitative estimates of cell populations; therefore, the above tick boxes do not apply. Trypsinized TSCs were pelleted, and the media was replaced with 1:1 TS base to RPMI. The cell suspension was then strained with a 40uM strainer.
Instrument	FACSARIA III, BD Biosciences
Software	BD FACSDiva Software (8.0.1 build 2014 07 03 11 47), Firmware (version 1.3)
Cell population abundance	Cell population abundance was not evaluated in this study.
Gating strategy	The nozzle for sorting was 130uM and speed was set to "flow rate 1". Cells expressing GFP intensities of the top 2-6% were considered positive and the bottom 2% were negative. These were single cell sorted and clones were then genotyped to confirm the mutant or wild-type genes. A reference negative was also used for calibration from cells that did not undergo the transfection but were seeded into the 6-well plate at the same time.

☒ Tick this box to confirm that a figure exemplifying the gating strategy is provided in the Supplementary Information.
